# Natural Compounds in Oral Microbiota Modulation and Caries Prevention: A Systematic Review

**DOI:** 10.3390/dj13110518

**Published:** 2025-11-05

**Authors:** María del Pilar Angarita-Díaz, Lilia J. Bernal-Cepeda, Jéssica María Sarmiento-Ordoñez, Yohan Yañez-Navas, Karen Garcia-Plazas, Hermann Gutierrez-Reyes, Laura Correa-Guataquira

**Affiliations:** 1 GIOMET Group, Faculty of Dentistry, Universidad Cooperativa de Colombia—Campus of Villavicencio, Villavicencio 500001, Colombia; yohan.yanez@campusucc.edu.co (Y.Y.-N.); karen.garciaplaza@campusucc.edu.co (K.G.-P.); hermann.gutierrezr@campusucc.edu.co (H.G.-R.); laura.correaguata@campusucc.edu.co (L.C.-G.); 2 IBAPO Group, Faculty of Dentistry, Universidad Nacional de Colombia, Bogotá 111321, Colombia; ljbernalc@unal.edu.co; 3 Grupo de Investigación, Innovación y Desarrollo Farmacéutico en Odontología, Faculty of Dentistry, Universidad Católica de Cuenca, Cuenca 010101, Ecuador; jsarmientoo@ucacue.edu.ec

**Keywords:** dental caries, microbiota, natural products, oral health

## Abstract

**Background/Objectives:** Certain components of natural products help maintain the oral microbiota balance, thereby promoting oral health. This study aimed to identify natural components with anticariogenic properties by analyzing evidence from in vivo studies. **Methods**: A systematic review was conducted in accordance with the Preferred Reporting Items for Systematic Reviews and Meta-Analyses (PRISMA) guidelines. The literature search was performed across multiple databases and included English-language studies published between 2013 and 2025. The review included intervention and comparative studies that examined the effects of dietary habits involving natural components in participants of any age, with or without dental caries. **Results**: A total of 77 studies were included in the review, most of which were clinical trials conducted in pediatric populations. To assess the impact of the interventions, most studies measured outcomes such as *Streptococcus mutans* levels, dental caries incidence, and salivary pH, among other parameters. The most frequently studied components included probiotics, plant extracts, sugar substitutes, propolis, arginine, dairy products, among others. Significant effects were most reported on biological risk factors (72.8%). In addition, 16.9% of the studies reported a statistically significant reduction in clinically diagnosed dental caries. **Conclusions**: This review identified preliminary evidence suggesting that certain natural compounds may play a role in modulating cariogenic factors. However, further high-quality studies are needed to strengthen the evidence base and confirm these findings. The protocol for this review was registered on the Open Science Framework platform.

## 1. Introduction

Over 700 bacterial species have been identified in the oral cavity, along with various species of fungi, viruses, and protozoa [1,2,3,4,5]. The presence and interactions of these microorganisms are influenced by factors such as the host’s immune response, systemic conditions, hygiene habits, diet, and antimicrobial use [6]. In response to these changing factors, the microbiota demonstrates both resistance and resilience. These properties can be strengthened by timely interventions that address early signs of disease [7,8].

When the microbiota is exposed to factors that disrupt the oral ecosystem, dysbiosis occurs. This condition is characterized by an imbalance favoring microorganisms with virulence factors promoting disease [9,10,11]. For example, dental caries—mainly triggered by excessive intake of fermentable carbohydrates—encourage the proliferation of cariogenic bacteria. These bacteria produce organic acids that demineralize tooth enamel, tolerate acidic environments, and synthesize extracellular polysaccharides from sucrose, facilitating colonization and dental biofilm formation [12,13,14].

Naturally occurring components with anticariogenic properties may contribute to rebalancing the oral microbiota through various mechanisms [15,16]. These include stimulating salivary flow, enhancing buffering capacity, and activating innate immune responses. Other effects involve inhibiting dental biofilm formation and cariogenic microorganisms (antimicrobial activity), promoting beneficial microbial species (prebiotic effect), and modulating the adaptive immune response [15,16].

However, most of these components—including plant extracts (e.g., leaves, seeds, flowers, and fruits) [17], dairy products [18], vitamins [19], probiotics [20], and sugar substitutes [21]—have primarily been studied under in vitro conditions or in animal models. Understanding their effectiveness in humans—specifically in reducing dental caries and modulating biological risk factors—is crucial. This knowledge will help identify which components could be incorporated into preventive strategies, such as nutritional guidance or other non-pharmacological interventions aimed at improving oral health.

This systematic review aims to address these gaps by focusing exclusively on in vivo human studies of natural anticariogenic components. It evaluates both clinical caries outcomes and relevant biological risk markers to better identify which agents may be effective under physiologically real conditions in humans.

## 2. Materials and Methods

### 2.1. Search Strategy

A systematic review was conducted and reported following the Preferred Reporting Items for Systematic Reviews and Meta-Analyses (PRISMA) guidelines [22] to ensure a transparent and reproducible search strategy for clinical studies evaluating naturally derived components for the prevention of dental caries and associated risk factors. Additionally, the Peer Review of Electronic Search Strategies (PRESS) framework was employed to validate the completeness and accuracy of the search strategy (conducted by Y.Y.-N and M.d.P.A.-D). The review protocol was registered on the Open Science Framework platform (DOI: 10.17605/OSF.IO/KF94A). No amendments were made to the registered protocol.

### 2.2. Eligibility Criteria

English-language studies published between 2013 and 2025 were included if they involved human participants of any age and assessed the effects of natural components on clinically diagnosed dental caries or related biological risk factors. Eligible designs included randomized and non-randomized clinical trials, longitudinal interventions studies, and observational studies. Exclusion criteria comprised studies conducted in vitro, in silico, or in animal models; grey literature; reviews; editorials; letters; unpublished studies, or expert opinions. Based on the PICO format, the following criteria were established: Population: Systematically healthy individuals of any age, not undergoing antibiotic treatment, with either caries-free or with measurable dental caries indices, with or without risk factors such as *S. mutans* quantification or dental biofilm index. Interventions: Naturally derived components with anticariogenic properties that can be ingested. Comparators: Placebo, other types of components, or no intervention. Outcome: Variables that assess the anticariogenic capacity (statistically significant effect). The included studies were grouped based on the type of natural components used in the interventions, and the study design.

### 2.3. Data Sources and Search Strategy

The databases consulted were PubMed, Scopus, Ovid, J-Stage, BVS, and Google Scholar. The review was conducted in November 2023, and updated in October 2025 using Medical Subject Headings (MeSH) keywords, including “dental caries,” “food,” “beverages,” “meals,” and “snacks,” as well as non-MeSH terms such as “anticariogenic,” “caries-free,” “antimicrobial,” and “cariostatic”. These keywords were combined with Boolean operators such as AND and OR (Appendix A). A supplementary manual search (conducted by M.d.P.A.-D) was also performed based on the components identified in the initial search.

### 2.4. Study Selection Process and Quality Assessment

#### Study Selection

The screening phase was conducted using the Rayyan Artificial Intelligence system, where the selection process was performed independently through the “Blind On” tool (Y.Y.-N, K.G.-P., L.C.-G, and H.G.-R.). In the event of discrepancies among the four reviewers, a fifth peer reviewer (M.d.P.A.-D adjudicated the final decision. In the initial phase, articles were selected based on their titles, abstracts, and full texts. In the second phase, data from studies that met the eligibility criteria (author, year, participants, components, frequency, duration, follow-up variables, and outcomes) were compiled in an Excel spreadsheet (Y.Y.-N, K.G.-P., L.C.-G, and H.G.-R.) and verified by M.d.P.A.-D and LB. Due to clinical and methodological heterogeneity among the studies (type of components, population, sample size, follow up duration), a meta-analysis was not appropriate. The findings were summarized after organizing key study characteristics for descriptive synthesis. To determine study eligibility for each synthesis, we tabulated the characteristics of all included studies, such as intervention type, frequency, follow up duration, comparator, and outcome measures. For studies reporting multiple interventions or outcomes, only data relevant to the predefined synthesis criteria were included. Additionally, the mechanisms of action of each component and their effectiveness, as reported in the literature, were investigated.

### 2.5. Methodological Quality

The methodological quality of the studies was first assessed using the Joanna Briggs Institute critical appraisal tool (JBI) [23], where the level of bias was evaluated based on four possible responses: compliant, non-compliant, unclear, and not applicable. Subsequently, to classify the grade of recommendation for each selected study, the Grading of Recommendations Assessment, Development, and Evaluation system [24] was used, categorizing the evidence as high, moderate, low, or very low quality. This process was independently performed by M.d.P.A.-D, L.J.B.-C., and J.S.-O. (Appendix B).

## 3. Results

### 3.1. Characteristics of the Included Studies

**Figure 1:**
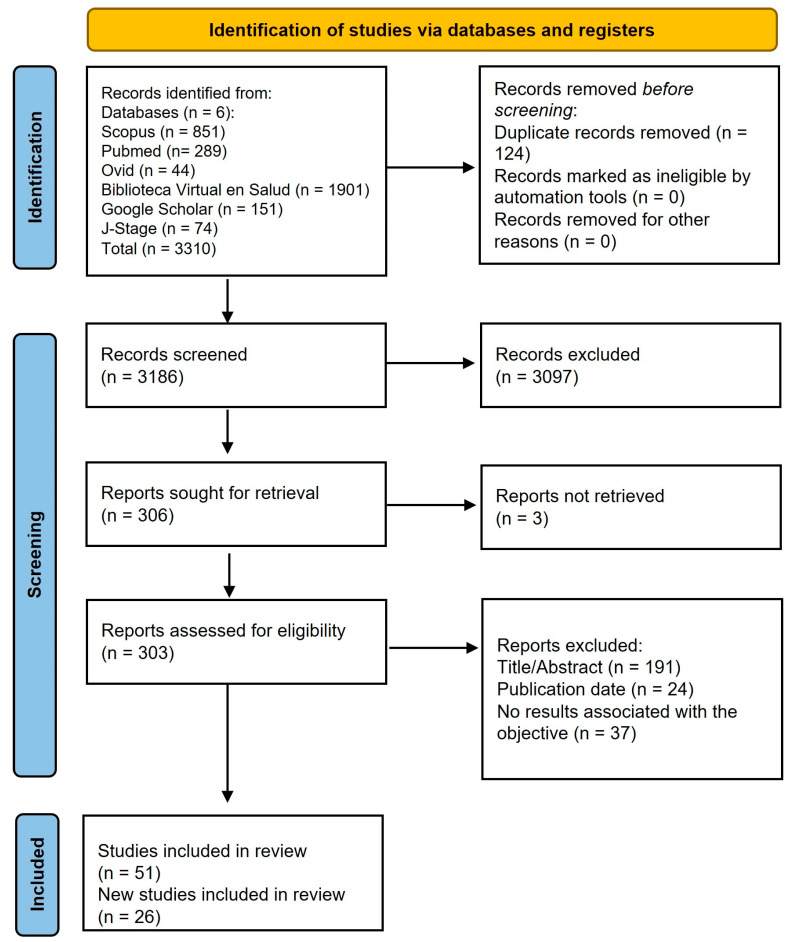
Flow diagram of literature search process.

After removing duplicates, a total of 3310 records were screened for eligibility (Figure 1). Of these studies, 51 that met the inclusion criteria were selected, and 26 additional studies identified through the manual search were included (Figure 1). The majority of the studies were clinical trials (80.5%) (Table 1). The most frequently evaluated components were probiotics (24.7%) (Table 1), particularly strains from the genus *Lactobacillus* species, followed by *Bifidobacterium*, *Limosilactobacillus*, and *Streptococcus* (Table 2). The second most studied component was plant extracts (22.1%), sugar substitutes (9.1%), followed by propolis (6.5%), arginine (6.5%), dairy products (5.2%), among others (Table 1). The variables used to assess anticariogenic effects included the clinically diagnosed dental caries (15%) and biological risk factors (85%) (Table 1). Furthermore, several studies have assessed the adverse effects and acceptability of the products.

The effects of the components were evaluated through various routes of administration, doses, and concentrations. The most common route included gum (15.6%), mouthwash (7.8%), tablet (6.5%) milk (5.2%) and varnish (5.2%) (Table 1). In total, 12,548 participants were included, with the majority falling within the age range of 6–12 years (childhood), followed by those between 12 and 18 years (adolescence), and 14–26 years (youth) (Table 2). Given the substantial heterogeneity in intervention components, dosing regimens, treatment durations, study populations, and outcome measures precluded the conduct of a meta-analysis.

### 3.2. Effects of the Anticariogenic Compounds

72.8% of the studies demonstrated significant effects on biological risk factors, in addition, 16.9% reported significant effects on clinically diagnosed dental caries (Table 1). Among the components demonstrating significant effects on clinically diagnosed dental caries were probiotics such as *L. rhamnosus* SP1 *L. reuteri* ATCC55730, *L. paracasei* SD1; synbiotic (probiotic and arginine); xylitol; toothpaste with arginine combined with dicalcium phosphate or with calcium carbonate; propolis; vitamin D; milk and vegetables (Table 2).

In biological risk factors, we identified components (probiotics, arginine, propolis, plant extracts, sugar substitutes, cherry or cranberry juice) with a significant effect on the decrease in *S. mutans* in saliva. Regarding salivary pH, the majority of the studies assessing this variable reported favorable outcomes, with probiotics, propolis, arginine, vegetable extracts, and dairy products being the most notable components. As for the dental biofilm index, most studies found a significant reduction, particularly with probiotics, propolis, vegetable extracts, apple chewing, and sugar substitutes like xylitol. Other beneficial effects, although less frequently reported, included increases in salivary flow, enhanced buffering capacity, and the production of nitrate, ammonium, and urea, as well as the arginolytic capacity of the microbiota. Regarding the adverse effects, a few studies (conducted with yogurt, milk, and sugar substitutes) reported mild adverse events, such as abdominal discomfort, headache, and allergic reactions (Table 2).

### 3.3. Quality of the Studies

The majority of the randomized, quasi-experimental, cross-sectional analytic, and cohort clinical studies (76.6%) met all or most of the criteria for the level of bias according to the tool used (Appendix B). Regarding the degree of recommendation, most studies were classified as having a moderate level (45.5%), followed by low (26%) and high (23.4%) levels (Table 1, Appendix B).

## 4. Discussion

Probiotics are the most extensively studied components; however, few studies have included long-term follow up and caries index assessment. Nevertheless, it is important to highlight that, depending on the strain of the probiotic used, a favorable effect on clinically diagnosed dental caries has been observed. In this review, the bacterial strains found to have favorable effect on caries indices included *L. rhamnosus* SP1 [38], *L. reuteri* ATCC 55730 [30], *L. paracasei* SD1 [35]. These findings support the potential of probiotics as a promising therapeutic strategy, particularly in children, as demonstrated in meta-analysis [20,102]. In contrast, other bacteria, such as those from the *Bifidobacterium* genus [26], do not exhibit the same effect, emphasizing the importance of selecting the correct strains, as well as determining the appropriate dosage, treatment duration, application vehicle, and considering interactions with other microorganisms and/or the host [103].

Some mechanisms of action of these beneficial microorganisms include the production of active metabolites (such as bacteriocins, hydrogen peroxide, and enzymes), inhibition of cariogenic microbial biofilm or adhesion, and competitive colonization against pathogens, competition for nutrients, and regulation of the immune system [104]. The other bacteria studied for dental caries control include *Streptococcus* strains, such as *S. dentisani*. Beyond its antimicrobial activity and prevalence in the oral cavity, this bacterium metabolizes arginine to produce ammonia, thereby contributing to the stabilization of dental biofilm pH [105].

Another component identified as impacting caries reduction is the amino acid arginine, which is considered a prebiotic compound in the oral cavity due to its ability to stimulate alkaline-producing bacteria [106]. This review identified L-arginine in reducing caries indices as reported in a two-year follow up study [67].

Tea is another component with favorable effects on dental caries-related indices, primarily due to its polyphenols, such as catechins, epigallocatechin-3-gallate, and epicatechin-3-gallate. These compounds possess antimicrobial properties, interfere with dental biofilm formation, and inhibit the glucosyltransferase activity of *S. mutans* [107]. In addition, tea contains amino acids, caffeine, and minerals such as fluoride, calcium, and phosphorus. However, the current evidence is insufficient to support the use of tea as a first-line treatment for caries [108]. In our review, the primary form of tea administration was through mouthwashes; however, chewing gum was also used for up to 2 years [54], showing a significantly favorable effect on caries reduction with no reported adverse effects. The effect of routine tea consumption remains controversial due to its potential erosive impact on enamel [109], although there is no conclusive evidence to support this claim [110].

Polyphenols are also found in other agents with a favorable impact on dental caries, such as licorice (*Glycyrrhiza glabra*), which contains flavonoids and isoflavonoids, as well as other phytocompounds like the triterpenoid glycyrrhizin, known to inhibit *S. mutans* glucosyltransferase. It also provides essential minerals (such as calcium) and vitamins (including biotin and niacin) [111]. Studies have demonstrated the stable antimicrobial capacity of licorice, with no adverse effects reported [112]. In this review, licorice extract was primarily used, delivered through mouthwashes and food-based products such as lollipops, offering an appealing route of administration for children and adolescents.

Sugar substitutes such as xylitol, sorbitol, erythritol, stevia, and maltitol [21] have been incorporated into food products like chewing gum, candies, milk, and cookies. Xylitol, a naturally occurring 5-carbon polyol, has been the most studied sugar substitute for its effect on the incidence of dental caries. It demonstrates significant favorable effects, particularly when used in chewing gum, as chewing stimulates saliva production, enhancing enamel remineralization [113,114]. Additionally, xylitol is non-fermentable by oral microbiota, thereby reducing acidogenic potential. However, some studies have reported adverse effects in participants with gastrointestinal issues. It is therefore recommended to limit the dosage to a maximum of 5 g, taken three times daily, or 7.5 g once daily [115].

Other components significantly affecting caries incidence include vitamin D, cranberry rinse, milk, and vegetables. Children and adolescents with insufficient vitamin D levels (<50 nmol/L) have a significantly higher probability of developing dental caries (odds ratio [OR]: 1.13–2.57). In contrast, individuals with sufficient levels (≥50 nmol/L) demonstrate a protective effect (OR in children: 0.80 and OR in adolescents: 0.59). This finding underscores the importance of improving vitamin D levels. Vitamin D is primarily obtained through exposure to sunlight, although it can also be sourced from certain foods, such as some fish [116]. Its mechanism of action in oral health is primarily linked to bone metabolism [116], mineralization of hard tissues, odontogenesis, and the immune system [19].

Milk has been associated with a lower risk of caries across various age groups [18]—either alone or in combination with fluoride, probiotics, and sugar substitutes—as well as its derivatives, such as yogurt and cheese, with preliminary results. Vegetables are another component of interest due to their association with caries-free individuals, as reported by other studies [117]. These foods are rich in vitamins, antioxidants, and fiber [118]. Moreover, vegetables, particularly leafy greens and root vegetables such as beetroot, are rich in inorganic nitrates that are absorbed into the bloodstream and various organs, including the oral cavity, after ingestion. Once in the oral cavity, these nitrates are converted to nitrite [119,120] and, in some cases, to nitric oxide. This process has a beneficial effect on the oral microbiota by increasing the abundance of nitrate-reducing bacteria, such as *Neisseria* and *Rothia* species [120]. Additionally, vegetables help stabilize salivary pH and reduce lactate production following a sugar rinse, as lactate is utilized by nitrate-reducing bacteria [121].

Our review also identified interventions that significantly and favorably impact the biological risk factors associated with dental caries. However, although the interventions demonstrated antimicrobial activity by inhibiting *S. mutans*, it is not the only cariogenic bacterium [122] and is not always present in patients with caries. Furthermore, not all studies conducted adequate microbiological analyses to quantify this bacterium. Another frequently studied variable was salivary pH, with some interventions (probiotics, arginine, tea, licorice, dairy products such as cheese, and fennel seeds) significantly increasing the pH. This result is attributed to the reduction in cariogenic bacteria, thereby decreasing the production of organic acids that induce enamel demineralization [123,124]. High pH levels, along with elevated salivary flow rate and buffering capacity, help prevent dental caries by neutralizing the acids produced by cariogenic bacteria [125]. Finally, the dental biofilm index was significantly reduced with various interventions (probiotics, synbiotic, propolis, licorice, apple, and cocoa bean husk extract), contributing to a reduction in the presence of cariogenic bacteria and their organic acids in the dental biofilm.

This review highlights the identification of ingredients categorized as biotics, which include probiotics (various bacterial strains), prebiotics (arginine, nitrate, sugar substitutes, vegetable extracts), and a combination of both, known as synbiotics (probiotics + arginine). Biotics are gaining popularity as they are increasingly preferred by populations, and emerging evidence supports their role in oral health management [104].

To better identify the natural components that can effectively improve oral health, further high quality randomized controlled trials with adequate sample sizes, longer follow-up periods, and standardized protocols are needed. Although this review included a substantial number of studies, most exhibited moderate to low methodological quality, relied on indirect measures such as *S. mutans* quantification rather than clinically diagnosed dental caries, had small samples sizes, short durations, and potential risks of bias such as inadequate randomization and lack of blinding. Moreover, significant heterogeneity in interventions, and outcome measures limits the generalizability of the findings and prevented the performance of a meta-analysis.

Clinically, natural anticariogenic agents show emerging potential for integration into preventive oral health programs; however, their effective and safe application requires careful evaluation of optimal formulations, dosages, and delivery methods, supported by robust regulatory oversight. Future research should focus on addressing these gaps by conducting rigorously designed trials that evaluate both efficacy and safety, as well as exploring the development of functional foods incorporating these components. Such efforts will be essential to translate preliminary findings into evidence-based clinical practice and public health strategies.

## 5. Conclusions

Probiotics, arginine, propolis, plant-derived compounds such as tea and licorice, and sugar substitutes like xylitol, vitamin D, milk, and vegetables have shown preliminary protective effects against dental caries through various mechanisms of action. However, the clinical efficacy of these agents remains to be confirmed, as most studies included in this review were of low or moderate methodological quality. Adverse events, such as stomach aches, headaches, or allergies, were infrequently reported, mainly in studies involving dairy products or sugar substitutes. Therefore, further high-quality clinical trials are necessary to substantiate these initial findings and better evaluate safety profiles.

## Figures and Tables

**Table 1:** Frequency of key characteristics in included studies.

Compounds	Plant Extracts	Probiotic	Arginine	Sugar Substitutes	Synbiotics	Propolis	Dairy products	Vitamin D	Other Components	Comparison of Different Components	Total
Studies %(n)	22.0% (17)	24.7% (19)	6.5% (5)	9.1% (7)	2.6% (2)	6.5% (5)	5.2% (4)	3.9% (3)	13% (10)	6.5% (5)	100% (77)
Study design											
Clinical trial %(n)	19.5% (15)	22.0% (17)	6.5% (5)	9.1% (7)	2.6% (2)	6.5% (5)	1.3% (1)	2.6% (2)	5.2% (4)	5.2% (4)	80.5% (62)
Cuasi-experimental	2.6% (2)	2.6% (2)	-	-	-	-	1.3% (1)	-	6.5% (5)	1.3% (1)	14.3% (11)
Cohort	0	-	-	-	-	-	2.6% (2)	1.3% (1)	-	-	3.9% (3)
Cross sectional									1.3% (1)	-	1.3% (1)
Grade quality											
High	3.9% (3)	6.5% (5)	5.2% (4)	3.9% (3)	2.6% (2)	-		-	1.3% (1)	-	23.4% (18)
Moderate	11.7% (9)	10.3% (8)	1.3% (1)	5.2% (4)	-	3.9% (3)	5.2% (4)	3.9% (3)	3.9% (3)	-	45.4% (35)
Low	6.5% (5)	3.9% (3)	-	-	-	1.3% (1)	-	-	7.8% (6)	6.5% (5)	26.0% (20)
Very low	-	3.9% (3)	-	-	-	1.3% (1)	-	-	-	-	5.2% (4)
Routes of administration											
Yogur	-	2.6% (2)		-	-		-	-	-	-	2.6% (2)
Tablet	-	5.2% (4)	1.3% (1)	-	-		-	-	-	-	6.5% (5)
Ice cream	-	1.3% (1)		-	-		-	-	-	-	1.3% (1)
Drops	-	2.6% (2)		-	-		-	-	-	-	2.6% (2)
Curd	-	3.9% (3)		-	-		-	-	-	-	3.9% (3)
Cake	-	1.3% (1)		-	-		-	-	-	-	1.3% (1)
Milk	-	3.9% (3)		1.3% (1)	-		-	1.3% (1)	-	-	6.5% (5)
Gel	-	1.3% (1)		-	-		-	-	-	-	1.3% (1)
Oral powder	-	1.3% (1)		-	-		-	-	-	-	1.3% (1)
Topical application	-	1.3% (1)		-	-		-	-	-	-	1.3% (1)
Lozenge	-	-		-	2.6% (2)		-	-	-	-	2.6% (2)
Gum	3.9% (3)			5.2% (4)	-	2.6% (2)	-	-	1.3% (1)	2.6% (2)	15.6% (12)
Candy	-			1.3% (1)	-	-	-	-	1.3% (1)	-	2.6% (2)
Cookies	-			1.3% (1)	-	-	-	-	-		1.3% (1)
Toothpaste	-		5.2% (4)		-	-	-	-	1.3% (1)	-	6.5% (5)
Mouthwash	14.3% (11)				-	1.3% (1)	-	-	-	6.5% (5)	22.0% (17)
Varnish	-			-	-	2.6% (2)		-	-	-	2.6% (2)
Lollipop	2.6% (2)				-	-	-	-	-	-	2.6% (2)
Juice	-				-	-	-	-	2.6% (2)	-	2.6% (2)
No applicable	-				-	-	5.2% (4)	2.6% (2)	5.2% (4)	-	13.0% (10)
Outcome categories *											
Clinically diagnosed dental caries **	1.3% (2)	4.6% (7)	1.3% (2)	1.3% (2)	1.3% (2)	0.6% (1)	1.3% (2)	2.0% (3)	1.3% (2)	-	15.0% (23)
Biological risk factor ***	19.6% (30)	26.8% (41)	5.9% (9)	11.1% (17)	-	5.2% (8)	2.6% (4)	0.6% (1)	9.8% (15)	3.3% (5)	85.0% (130)
Results											
% Statistically significant. Clinically diagnosed dental caries	2.6% (2)	3.9% (3)	1.3% (1)	-	1.3% (1)	-	2.6% (2)	3.9% (3)	1.3% (1)	-	16.9% (13)
% Statistically significant. Biological risk factors	19.5% (15)	15.6% (12)	5.2% (4)	7.8% (6)	-	5.2% (4)	2.6% (2)	-	10.3% (8)	6.5% (5)	72.7% (56)
% Non-significant effect. Clinically diagnosed dental caries	-	2.6% (2)	-	1.3% (1)	1.3% (1)	1.3% (1)	-	-	-	-	6.5% (5)
% Non-significant effect. Biological risk factors	-	2.6% (2)	-	-	-	-		-	1.3% (1)	-	3.9% (3)

Outcome categories *: his value was calculated based on the total number of measured (n = 153) outcomes across all included studies, not merely on the count of studies. Clinically diagnosed dental caries **: ICDAS; Occurrence of caries; Caries transition; Tooth decay incidence (number of new dmft or DMFT). Biological risk factor ***: Quantification of Streptococcus mutans; Quantification of Lactobacillus; Salivary flow; Biofilm index; Salivary or Biofilm pH; Salivary buffering, Quantification of Secretory IgA; Measure total lactic acid; Oral microbiota composition; Concentration of hβD-3; Chemical analysis of biofilm; among others.

**Table 2:** In vivo studies evaluating anticaries compounds of natural origin.

Author/Country	Population	Frequency/Follow UP	Control	Outcome Measure Techniques	Results
Randomized Controlled Trials					
Intervention: Probiotics					
Poureslami et al., 2013 Iran [25]	47 oral healthy female students aged 15–17 years old	For 2 weeks (Cross-sectional cohort study): - G1: 100-g Espar (25 × 10^8^ Colony Forming Unit -CFU- of *Lactobacillus* per gram)	Control: - G2: 200-g yogurt (4 × 10^6^ CFU Lb per gram)	Unstimulated saliva: - Quantification of *Streptococcus mutans*-Sm-(Blood agar+Neomycin). - Calcium: (photometry method)	- G1: significant decrease in Sm. Significant increase in calcium content (*p* < 0.001) - G2: No significant effect on Sm (*p* > 0.05). Significant increase in calcium content (*p* < 0.001)
Taipale et al., 2013 Finland [26]	106 children aged 1–2 months	Twice daily, until the child reached 2 years of age. - G1: probiotic tablet [(*Bifidobacterium animalis* subsp. *lactis* BB-12 (5 × 10^9^ CFU)] + 100- or 300-mg xylitol	Controls: - G2: 100-or 300-mg xylitol tablet - G3: 100-or 300-mg sorbitol tablet	- Ocurrence of caries (International Caries Detection and Assessment System -ICDAS-) Biofilm sampling: - Quantification of Sm at the age of 2 years (Mitis salivarius Agar -MSA-) - Quantification of Sm at the age of 4 years (Dentocult^®^)	- No differences were detected between the study groups in the occurrence of enamel caries (*p* > 0.05) or obvious dentinal caries (*p* > 0.05). In addition, there was no significant effect on Sm (*p* > 0.05)
Chinnappa et al., 2013 India [27]	40 oral healthy participants aged 12–14 years old	For 7 days (Cross-sectional cohort study) - G1: 100-mL probiotic ice-cream - G2: 100-mL probiotic curd	Placebos: - G3: 100-mL regular ice cream - G4: 100-mL regular curd	- Quantification of Sm in saliva (MSA)	- All groups: significant decrease in Sm (*p* < 0.001) after 1 h - G1 and G2: significant decrease in Sm (*p* < 0.001) compared to the placebos (G3 and G4) after 7 days
Pinto et al., 2014 Brazil [28]	26 oral healthy participants aged 10–30 years old (mean age: 15 years).	For 2 weeks (Cross-sectional cohort study) - G1: 200-g probiotic yogurt (*B. animalis* subsp. *lactis* DN-173010)	Placebo: - G2: 200-g regular yogurt	Stimulated saliva and biofilm: - Total microorganisms (Blood agar) - Quantification of Sm (MSA + Bacitracin) - Quantification of Lactobacillus -Lb- (De Man Rogosa Sharpe agar -MRS-)	- G1 and G2: No significant effect on Sm or Lb, but a significant decrease in total microorganisms in the biofilm (*p* < 0.01) - G2: Stomach discomfort reported by one patient
Nishihara et al., 2014 Japan [29]	- Study 1: 64 participants (mean age: 24.8 ± 2.3 years old) - Study 2: 8 healthy participants	- Study 1: Once time: - G1: One tablet of *Ligilactobacillus salivarius* (formerly *Lactobacillus salivarius*) WB21 (6.7 × 10^8^ CFU and 280-mg xylitol per tablet) - G2: One tablet of *L. salivarius* TI 2711 (2.8 × 10^8^ CFU and 450 mg of xylitol per tablet) - G3: Ovalgen^®^ DC (antibodies against glucosyltransferase of Sm and 100-mg xylitol per tablet) - Study 2: for 2 weeks: - One tablet of *L. salivarius* WB21, three times per day	Placebo: - Study 1: G4: One Tablet of Xylitol (280-mg per tablet)	- Study 1: - Biofilm and stimulated saliva: Quantification of Sm (Dentocult^®^ SM Strip mutans) and Lb (Dentocult^®^ LB) - Salivary flow (Gum test) - Salivary pH - Salivary buffering capacity (CheckBuf test kit) - Study 2: - Quantification of Sm (MSA + bacitracin)	- Study 1: - G1: significant decrease in Sm. Significant increase in Lb - G2: significant decrease in Sm. Significant increase in Lb - G3: significant decrease in Sm. Significant increase in salivary buffering - Salivary flow and salivary pH: no significant differences between the four groups (G1–G4) - Study 2: - The intervention significantly decreased Sm
Stensson et al., 2014 Sweden [30]	113 children (mean age: 9.1 ± 0.3 years old in the probiotic group and 9.2 ± 0.4 years old in the placebo group)	- G1: Five drops of *Limosilactobacillus reuteri* (formerly *Lactobacillus reuteri*) ATCC 55730 (10^8^ CFU), for 4 weeks before the expected date of delivery, continued until the child was born, and five drops orally throughout the first year of life (thus 365 days)	Placebo: - G2: treated in the same way (drops without any bacteria)	At 9 years of age: - Ocurrence of caries Stimulated saliva and biofilm sampling: - Quantification of probiotic *L. reuteri* (MRS) - Quantification of Sm (MSA + bacitracin) and Lb (MRS) - Quantification of Secretory IgA -SIgA- - Gingival Bleeding - Biofilm index	- G1: 82% of the participants were caries-free, a significant difference from G2 (58%, *p* < 0.01). No significant effect on *L. reuteri*, Sm, Lb, salivary SIgA (*p* > 0.05) or biofilm index. The prevalence of proximal caries lesions was lower (*p* < 0.05) and there were fewer sites with gingivitis compared to G2 (*p* < 0.05)
Bhalla et al., 2015 India [31]	30 participants aged 12–14 years old	Once a day for 7 days: - G1: 200-g probiotic curd, once daily (*Bifidobacterium lactis* 12, Bb-12)	Placebo: - G2: 200-g regular curd	Unstimulated saliva: - Quantification of Sm (MSA + Bacitracin)	- G1: Significant reduction in Sm counts in saliva after 1 h and 7 days compared with G2 (*p* < 0.01)
Cortés-Dorantes et al., 2015 Mexico [32]	26 patients aged 4–6 years old with a high risk of dental caries [≥1500 Relative Light Units (RLU)]	For 2 weeks: - G1: ingested Probiora3^®^ oral tablets (*Streptococcus uberis* KJ2TM, *Streptococcus oralis* KJ3TM and *Streptococcus rattus* JH145TM)	Placebo: - G2: tablets with similar presentation but without probiotics or sugars.	Saliva film with a swab: - Luminescence assay: CariScreen^®^ and CariScreen Susceptibility Testing Swabs^®^	- G1: Significant reduction in Sm levels before and after probiotic suspension, but the reduction was greater than that in G2 (*p* < 0.05) - G2: Significant reduction in Sm levels
Srivastava et al., 2016 ** India [33]	60 oral healthy participants aged 20–25 years old	For 7 days: G1: Probiotic curd (*Lactobacillus acidophilus, B. animalis subsp. lactis*)	Placebo: - G2: normal Curd	Stimulated saliva: - pH value (pH meter) - Quantification of Sm (MSA + Bacitracin + potassium tellurite)	- G1: Significant increase in pH value (*p* < 0.05). Significant decrease in Sm (*p* < 0.05) - G2: Significant decrease of pH (*p* < 0.05). No significant effect in Sm (*p* > 0.05)
Koopaie et al., 2019 Iran [34]	40 oral healthy participants aged 15–73 years old (mean age: 41.67 ± 16.80 years)	For 1 week (cross-sectional cohort study): - G1: 70-g probiotic cake (*Bacillus coagulans*)	Placebo: - G2: regular cake	Unstimulated Saliva: - Quantification of Sm (MSA) - pH value (pH test strips)	- G1: No significant increase in Sm (*p* > 0.05) No significant effect on pH (*p* > 0.05) -G2: significant increase in Sm compared with baseline (*p* < 0.05). No significant effect on pH (*p* > 0.05) - No adverse effects
Piwat et al., 2020 Thailand [35]	487 children (mean age: 37.6 ± 9.2 months)	Once a day for 6 months: - G1: probiotic milk (*Lacticaseibacillus paracasei* (formerly *Lactobacillus paracasei*) SD1) - G2: probiotic milk (*L. paracasei* SD1) for 3 days a week and the placebo milk for the remaining 4 days	Placebo: - G3: milk without probiotics	- Caries transition, including caries progression and regression during the T0–T6, T6–T12, and T0–T12 periods - Modified visible dental biofilm index score	- G1 and G2: A decreased caries risk during T0–T6. Increased regressive surfaces were observed during T0–T12. After a 1-year follow-up, significant increases in caries regression were observed - Three children (1 in each group) presented allergic symptoms, which were skin rash or vomiting.
Ferrer et al., 2020 Spain [36]	50 subjects aged 18–65 years old	Dental splint for 5 min every 48 h, for 1 month: - G1: Buccoadhesive gel of probiotic (*Streptococcus dentisani* 2.5 × 10^9^ CFU/dose)	Placebo: - G2: Buccoadhesive gel of excipients without probiotics	- Quantification of *S. dentisani* in dental plaque and saliva samples - Plaque index (PI) and Gingival index (GI) - Salivary flow -Saliva electrolytes quantification -Measure total lactic acid and pH of saliva and plaque samples -Oral microbiota composition of dental plaque	- G1: Significant increase the abundance of *S. dentisani.* Species at significantly higher levels in the probiotic group: *Gemella morbillorum, Porphyromonas pasteri*, and *Kingella* sp. A progressive decrease in the biofilm index, which is more pronounced than in G2. Significant increase in salivary flow compared to G2 at 15 days. Significant increase in salivary calcium and ammonium concentrations. No effect on pH and lactic acid produced by biofilm -G2: A significant decrease in pH produced by biofilm values after 15 days of treatment
Sakhare et al., 2021 India [37]	57 children aged 6–12 years old	Two times a day for 21 days - G1: 100-g probiotic curd (*L. acidophilus* (La5) and *Bifidobacterium* lactis (Bb12)	Control: - G2: not given anything to consume	Stimulated saliva: - Quantification of *S. mutans* (Sm) (MSA) - Ph	- G1: No significant difference in Sm count after 7 days compared with G2 (*p* > 0.05). A significant decrease in Sm count at the end of 24 days in G1. There was no statistical difference in the salivary pH between the two groups
Sandoval et al., 2021 Chile [38]	42 children aged 2–3 years old	Daily, for 10 months: - G1: 150-mL 2% milk supplemented with 10^7^ CFU/mL of *Lacticaseibacillus rhamnosus* (formerly *Lactobacillus rhamnosus*) SP1	Placebo: - G2: 150-mL medium-fat milk	- Dental caries (ICDAS). - Concentration of hβD-3 in unstimulated saliva (ELISA test)	- G1: Significant decrease in hβD-3 levels (from 597.91 to 126.29 pg/mL, *p* < 0.05). No increase in carious lesions - G2: There was an increase in the number of teeth with carious lesions (dICDAS2-6 mft)
Janiani et al., 2022 India [39]	30 children aged 3–6 years old, with score < 2, for primary teeth (decayed, extracted, filled teeth -deft-) for permanent teeth (decayed, missing, filled teeth -DMFT-)	Once daily, for 7 days: - G1: 1 sachet (oral powder, tongue-dissolving, 2.7 billion CFU of *L. acidophilus* UBLA-34, *L. salivarius* UBLS-22, *L. rhamnosus* UBLR-58, *L. paracasei* UBLPc-35) - G2: 10-mL of probiotic milk (*Lactobacillus casei* Shirota)	Control: - G2: not given anything to consume	- Biofilm index (Silness–Löe) - Quantification of Sm in unstimulated saliva (MSA)	- G1: Significant reduction in biofilm score (*p* < 0.05) - G1 and G2: Significant reduction in Sm (*p* < 0.05). G1 had a greater reduction
Hasslöf et al., 2022 Sweden [40]	28 children aged 2–5 years old	For 12 months: - G1: 5 drops of the probiotic (100 million live bacteria of *L. reuteri* (DSM 17938 and *L. reuteri* DSM 17938 and ATCC PTA 5289)	Control: - G2: drops without probiotic bacteria	- Ocurrence of caries (ICDAS) - Gingival condition (bleeding-on-brushing) - Visible supra-gingival plaque on the buccal surfaces	- The recurrence of moderate and extensive lesions (ICDAS 3-6) was similar between G1 and G2 - Approximately 70% of children in G1 and G2 had visible plaque on their upper anterior teeth at 12 months. No beneficial effects on dental plaque or gingival inflammation - Findings were uncertain and inconclusive due to lack of power - No adverse effects
Staszczyk et al., 2022 Poland [41]	127 aged 3–6 years old (median age: 4.51 ± 0.94)	Two tablets per day, for 14 days: - G1: 10-mg thermally inactivated *L. salivarius* HM-6	Control: - G2: not given anything to consume	- Ocurrence of caries (ICDAS, dmft) - Visible Biofilm accumulation (Oral Debris component of the Simplified Oral Hygiene Index DI-S of OHI-S index)	- G1: The incidence and prevalence of caries were significantly lower in G1 compared with G2 (*p* < 0.001). No significant effect on the biofilm - G2: significant decrease in biofilm (*p* < 0.001) - No adverse effects
Intervention: Synbiotics -Probiotic and Arginine-					
Pørksen et al., 2023 Denmark [42]	288 children aged 5–9 years old (mean age: 7.2 years)	Daily, for 10 or 12 months - G1: lozenges of probiotics (*L. rhamnosus*, LGG^®^ (DSM33156) 1 × 10^9^ CFU, *L. paracasei* subsp. *paracasei, Lactobacillus casei* 431^®^ (DSM33451) 1 × 10^9^ CFU, arginine (20 mg ∼ 2%), and xylitol (627 mg)	- G2: lozenges without probiotics or arginine but with xylitol (671 mg)	- Clinical caries registration (ICDAS): weighted Δd/DICDAS1–2w/b,3–6m/M-s/S-f/F-s/S, unweighted ΔdICDAS3–6msf-s, weighted caries progression, weighted caries stagnation, weighted ΔDICDAS1–2w/bMSF-S, weighted ΔDICDAS3–6MSF-S, weighted caries regression. - Plaque index (PI) and Gingival index (GI)	- G1: Significantly lower ICDAS score (*p* < 0.01). No significant effect on biofilm and gingival index - No adverse effects
Pørksen et al., 2023 ** Denmark [43]	288 children aged 5–9 years old (19,950 tooth surfaces)	Daily, for 10 to 12 months: - G1: lozenges with arginine (2%), and 2 billion CFU of the probiotics (*Lacticaseibacillus rhamnosus*, LGG^®^, and *L. paracasei* subsp. *paracasei*) + 1450 ppm F- toothpaste	Placebo: G2: lozenge without probiotic or arginine + 1450 ppm F- toothpaste	Tooth decay incidence (ICDAS_0–6_, radiographically (R_0–6_)	- G1: No significant difference in results compared with G2 (*p* > 0.05), however: Less progression of caries (5.5%) in comparison with G2 (6.3%). More caries regression (3.5%) compared with G2 (3.2%). Fewer active lesions (15.3%)
Intervention: Sugar Substitutes					
Campus et al., 2013 Italy [44]	148 children aged 7–9 years (mean age, 8.3 ± 1.2 years), with the presence of two or three carious lesions in the permanent and/or primary dentition, and a salivary MS concentration of > 10^5^ CFU/mL	Chewing gum five times/day for 6 months - G1: two pellets of xylitol gum [(xylitol (36.6%), sorbitol (17.7%), maltitol (9.7%), mannitol (7.1%)]. The total daily intake of xylitol was 11.6 g	Placebo: - G2: non-xylitol chewing gum (isomalt (30.0%), sorbitol (17.7%), maltitol (16.3%), mannitol (7.1%), gum base, flavors, humectants, food color, acidity regulator and glazing agents)	- Quantification of Sm and Lb (CTR bacteria by Ivoclar-Vivadent) (stimulated saliva) - Caries registration (both initial and manifest caries were scored)	- G1: significantly lower increment in the number of new carious lesions (1.43% for manifest lesion and 2.86% for initial lesions) - G2: increment in the number of new carious lesions (10.26% for manifest lesion and 16.66% for initial lesions) - G1 and G2: No statistically significant decrease in the percentage of subjects with an MS of > 10^5^ CFU/mL (*p* > 0.05). Significant reduction in Lb at the 2-year examination (*p* < 0.05) - No adverse effects
Runnel et al., 2013 Estonia [45]	374 children aged 7–8 years old	0.7 g candies 3 times per day for 3 years (daily consumption: 7.5g): - G1: erythritol candy (90%) - G2: xylitol candy (90%)	Control: - G3: candy of sorbitol (90%) An additional comparison group (endpoint group)	- Quantification of Sm (Dentocult SM) and Lb (Dentocult LB) (stimulated saliva and biofilm) - Fresh weight of biofilm - Chemical analysis of biofilm (sugars, organic acids, polyiols, calcium, glucose, glycerol)	- G1: Significant reduction in Sm, in dental biofilm compared to G2 y G3 (*p* < 0.05). Significantly lower levels of acetic acid and propionic acid than xylitol and sorbitol (*p* < 0.05) - There was no change in salivary Lb levels in all groups
Chi et al., 2017 ** Perú [46]	127 children (mean age: 6.9 ± 1.8–8.0 ± 2.3 years old)	For 9 months: - G1: Millk + xylitol (8g in 200-mL milk once/day) - G2: Milk +xylitol (4g in 100-mL milk twice/day)	Active treatment control: - G3: Milk + sorbitol (8g in 200-mL milk once/day). - G4: Milk + sorbitol (4g in 100-mL milk twice/day) Control: - G5: Milk + sugar (8g in 200-mL milk once/day).	- Quantification of Sm and *S. sobrinus* (MSA + bacitracin + potassium tellurite) (biofilm) - Tooth decay incidence (number of new dmft or DMFT)	- G1 and G2: significant decrease in Sm compared with sugar (*p* < 0.05). No difference between G3 and G4 (*p* > 0.05). No difference in tooth decay incidence between groups - Side effects: 8.5% reported symptoms such as stomachache, headache, and/or vomiting [8g sorbitol once/day group (14.3%), 4-g xylitol twice/day group (11.1%), 4-g sorbitol twice/day group (6.7%), 8-g xylitol once/day group (6.5%), sucrose group (3.6%)] - No adverse effects
Aluckal 2018 India [47]	60 children aged 12–15 years old, with salivary Sm level equal to or more than 10^5^ CFU and DMFT score ≥ 3	Chewing one gum for 5 min, twice daily for 28 days (+4 gums): - G1: xylitol chewing gum - G2: polyol chewing gum	Control: - G3: with no chewing gum	- Quantification of Sm (MS + potassium tellurite) (stimulated saliva). - Acceptability	- G1: significant reduction in Sm compared to G3 (*p* < 0.05) - G2: significant reduction in Sm (*p* < 0.05) - G1 and G2: Very good (18.3%) and good (26.7%) acceptance
Abdelwahab et al., 2018, Egypt [48]	42 children aged 3–12 years old, with high caries risk (DMFT/dmft/deft ≥ 3)	Chewing one gum pellet twice a day, for 3 weeks: - G1: Xylitol gum (Mentos^®^ White Chewing Gum) (0.28-g xylitol per day) - G2: sugar-free polyol gum (Mentos^®^ Juice Blast Chewing Gum)	No placebo/control group	- Quantification of Sm and Lb (CRT^®^ kit by Ivoclar-Vivadent) (stimulated saliva)	- G1: maximum effect in reducing Sm, particularly in children aged 3–6 years, followed by children aged 6–9 years. Maximum effect of reducing Lb in children aged 3–6 years and the worst effect in children aged 6–9 years - G2: maximum effect in reducing Lb in children aged 6–9 years and the worst effect in reducing Sm in children aged 9–12 years
Cocco et al., 2019 Italy [49]	264 children aged 6–9 years old, with one or two initial carious lesions in the enamel (ICDAS 2–3) but without a manifest lesion in the dentin (ICDAS 5–6), and Sm ≥ 10^5^ CFU/mL.	Twice a day for 42 days: - G1: cookies with stevia - G2: cookies with maltitol	Control: - G3: cookies with white sugar	- Oral clinical evaluation: Carious lesion (ICDAS), bleeding on probing. - Quantification of Sm and Lb (CTR bacteria by Ivoclar-Vivadent) (stimulated saliva). - Interproximal biofilm pH (pH indicator strips): before and after a mouthrinse with a solution containing 10% sucrose.	- G1: significant decreases in Sm and Lb (*p* < 0.05). Increase in the minimum and maximum pH and reduction in the pH drop (*p* < 0.01). Low probability of developing new caries compared with the other groups (*p* < 0.01) - G2: significant decrease in Lb (*p* < 0.05). Increase in the maximum pH (*p* < 0.01) -G3: No effect on the bacterial count. Increase in maximum pH and pH drop was observed (*p* < 0.01) - G1, G2, G3: significant decrease in bleeding score (*p* < 0.05) - No adverse effects
Akgül et al., 2020 Turkey [50]	147 healthy oral participants (mean age 23.43 ± 2.3 years old)	Chewing gum 3 times a day, for 3 weeks - G1: 5.4 g xylitol daily	Placebo: - G2: non-xylitol chewing gum	- Oral clinical evaluation (Gingival Index and Plaque Index) - Analysis of cytokines (TNF-α, IL-6, IL-8) (Enzyme-linked immunosorbent assay) (Unstimulated saliva) - Quantification of Sm gtfB gene expression [(quantitative real-time PCR (qPCR)]	- G1: significant reduction in biofilm (*p* < 0.05) and gingival scores (*p* < 0.001). Significant reduction in cytokine (*p* < 0.001) and gtfB gene expression (*p* < 0.001)
Intervention: Plant Extracts					
Tea					
Neturi et al., 2014 India [51]	30 subjects aged 20–25 years old (mean age 22.4 ± 1.75)	Rinse with 10 mL of respective solutions for one minute (the participants were exposed to all the three rinses with a wash out period of seven days between intervention): -G1: Green tea	Positive control: - G2: Chlorhexidine -CHX- Negative control: - G3: Water	- Quantification of Sm (Chocolate agar)	- G1: significant decrease in Sm in G2 (*p* < 0.01) and G1 (*p* < 0.01)
Thomas et al., 2016 India [52]	30 children aged 4–6 years old (mean age: 5 ± 0.69) with severe early childhood caries (S-ECC)	Rinse for 1 min, for 2 weeks: - G1: 5-mL green tea extract (0.5% phenolic compound using double distilled water)	Control: - G2: CHX (0.2%)	- Quantification of Sm (MSA+bacitracin), Lb (MRS)*, Candida* (HiCrome) (unstimulated saliva) - Acceptability	- G1 and G2: significant decrease in Sm and Lb (*p* < 0.001) - G1: Non-significant decrease in *Candida*. The majority of the participants had a positive response to flavor (66.7%), smell (55.3%); and willingness to rinse (66.7%) - G2: non-significant increase in *Candida*
Prihastari and Putri, 2022 Indonesia [53]	42 children aged 7–8 years old	Chewing gum for 2–3 min, for 3 weeks: - G1: chewing black tea candy containing sorbitol	Placebo - G2: candy without black tea	- Unstimulated salivary pH (pH meter) - DMF-T index score	- G1: Significant increase in pH (*p* < 0.001) - G2: Non-significant change in pH (*p* > 0.05)
Tao et al., 2013 ** China [54]	168 children aged 8–9 years old (mean age 4 ± 0.5 years)	Twice a day for 8 min, for 2 years: - G1: Chewing gum with tea polyphenol	Positive control: - G2: chewing gum without tea polyphenol Negative control: - G3: not receive any chewing gum	- Tooth decay incidence (DMFT and DMFS score)	- G1: Significantly lower DMFS (*p* < 0.05). Higher proportion of children with DMFT index ‘0’ - No adverse effects
Hedge and Kamath, 2017 ** India [55]	71 children aged 8–12 years old (mean age (11.2 ± 0.93 years) with at least four dmft ≥ 4	Rinse for 1 min using 10-mL, twice daily for 2 weeks: - G1: Green tea extract (0.5%)	Positive control: - G2: CHX (0.12%) - G3: combination mouth rinse (fluoride -F- and CHX)	- Quantification of Sm (MSA + bacitracin) and Lb (MRS) (stimulated saliva)	- G1: significant decrease in Sm and Lb (*p* < 0.001) - G2: significant decrease in Sm and Lb (*p* < 0.001), but significantly lower as compared to G1 and G3 - G3: no difference from G1
Kamath et al., 2021 ** India [56]	50 children aged 8–12 years old (mean age: 21.77 ± 2.06) with four or more (decay component) of dmft index	Rinse for 1 min using 10-mL, twice daily for 2 weeks - G1: Green tea extract (0.5%)	Positive control - G2: CHX (0.12%)	- Quantification of Sm (MSA + bacitracin) (biofilm)	- G1: significant decrease in Sm (*p* < 0.001) with similar results to G2 (*p* > 0.05)
Intervention: Licorice					
Jain et al., 2013 ** India [57]	60 children aged 7–14 years old with at least 5 active decayed tooth surfaces and poor oral hygiene	Rinse for 1 min using 10-mL: - G1: aqueous licorice extract (1.5 g/10 mL saline solution) - G2: ethanolic licorice extract (375 mg/10 mL)	- G3: CHX (0.0156%)	Unstimulated saliva: - pH value (test paper) - Quantification Sm (MSA + bacitracin)	- G1: Significant decrease in Sm (*p* < 0.001). An immediate rise in salivary pH - G2: Significant decrease in Sm (*p* < 0.001). Significant difference in Sm from G3 (*p* < 0.01). Rise in pH at the immediate post-rinse interval - G3: Significant decrease in Sm (*p* < 0.001). A drop in pH at the immediate post-rinse interval
Almaz et al., 2017 Turkey [58]	97 participants aged 5–11 years old (mean age: 6.8 ± 2.0–7.5 ± 1.8), caries-free or high-caries-risk	Twice a day for 10 days: - G1: Sugar-free licorice root extract lollipop	Placebo: - G2: Sugar-free lollipops	- Quantification of Sm (Dentocult SM Strip Mutans test) (stimulated saliva)	- G1: significant decrease in Sm (*p* < 0.05) - G2: Non-significant reduction in Sm
Kim and Nam, 2021 ** Korea [59]	60 oral healthy participants (mean age: 44.27–45.53 years old)	Rinse using 15-mL, for 5 days: - G1: licorice extract (*Glycyrrhiza uralensis*, 1 mg/1 mL)	Placebo: - G2: Saline solution	- Acid production capacity of the biofilm (Cariview test) - Microbiological analysis (Sm, *S. sobrinus, Actinomyces viscosus*) of gingival sulcus (qPCR)	- G1: significant decrease in acid production rate (*p* < 0.05). - Low risk was increased. - Effective reduction of bacteria - G2: No significant effect on acid production. High risk was increased
Helmy et al., 2021 ** Egypt [60]	46 participants aged 18–15 years old (mean age 31.65 ± 9.61–34.04 ± 11.15 years)	Rinse for 1 min using 10-mL, once a day for 1 week: - G1: Licorice mouthwash (16mg/mL)	Positive Control: -G2: CHX	Unstimulated saliva: - Quantification *Sm* (MSA + bacitracin + potassium telurite) - pH value (pH meter) - Biofilm index (Silness y Löe index)	- G1: significant decrease in Sm (*p* < 0.001). No difference with G2 (*p* > 0.05). - Significant increase in pH immediately after (*p* < 0.01). - Significant decrease in biofilm (*p* < 0.05), but CHX was better
Kamal et al., 2021 ** Egypt [61]	63 participants aged 18–35 years old (mean age 29.61 ± 4.51) at high risk of caries	Rinse for 1 min using 10-mL, once a day for 1 week of each month. This regimen was repeated monthly for a period of 1 year: - G1: Gum Arabic extract (6.25%) - G2: Licorice extract (12.5%)	- G3: CHX- (0.12%)	- Quantification Sm (MSA + bacitracin) and Lb (MRS) (stimulated saliva) - Clinical Examination (DMF)	- G1: DMF was significantly lower than that of G3. Significant decreases in Sm and Lb counts (*p* < 0.001). No adverse effects - G2: DMF was significantly lower compared to G3. Significant decreases in Sm and Lb counts (*p* < 0.001). No adverse effects G3: significant increase in DMF score (*p* < 0.001). Significant increase in Sm and Lb counts (*p* < 0.001). Multiple oral side effects
Intervention: Cocoa Bean Husk Extract					
Kibriya et al., 2023 India [62]	80 children aged 7–12 years old	Rinse with 10-mL for 30 s, for 2 weeks: - G1: 0.1% cocoa bean husk extract -CBHE-with distilled water - G2: 0.1% CBHE with Ringer’s lactate - G4: 0.1% CBHE with normal saline	- G3: 0.12% CHX - G5: 0.05% sodium fluoride (NaF)	- A Simplified Oral Hygiene Index (OHI-S) -Biofilm score Unstimulated saliva: - Salivary pH - Quantification Sm (MSA)	- G1, G2, G3, and G4: proved to be better anti-plaque agents than G5 - The mean colony count showed a maximum reduction in G4 followed by G1 - All groups except G2 showed a significant increase in pH - A major proportion of subjects in all the groups did not experience any unpleasant sensations (vomiting, excessive, salivation)
Intervention: Cranberry Extract					
Bansal et al., 2024 India [63]	280 children aged 8–12 years old	Rinse with 10-mL, once a day, for one minute, for 4 weeks: G1: non-dialyzable material (NDM) containing cranberry mouth rinse	Positive control: Sodium fluoride (F-MR) mouth rinse.	- Quantification Sm (MSA + bacitracin) and Lb (MRS) (dental biofilm)	- G1: Significant decrease in Sm (*p* = 0.001). No difference with positive control (*p* > 0.05).
Intervention: Costus					
Salem et al., 2025 Egypt [64]	75 children aged 6–13 years old	Rinse with 5-mL, twice per day for 1 week: - G1: 5% Indian Costus - G2: 10% Indian Costus - G3: 15%% Indian Costus	Negative control: tap water Positive control: CHX	Quantification Sm (MSA + bacitracin) (non-stimulated saliva)	- G1, G2, G3 and positive control: Significant decrease in Sm (*p* < 0.001). G3 showed the greatest reduction.
Intervention: Arginine					
Vuletic et al., 2013 ** Croatia [65]	117 participants (mean age 21.7 ± 1.8 years old)	Single daily dose of 3 g: - G1: three tablets of L-arg	Placebo: - G2: three tablets without L-arg	One hour after the intervention: - Unstimulated saliva pH (pH meter) -Salivary flow rate - Salivary urea concentration SaU (UV enzymatic urease-glutamate dehydrogenase method)	- G1: Significantly reduced urea concentration (*p* < 0.05), but similar to G2 (*p* > 0.05). Significant increase in pH (*p* < 0.001) - G2: No significant increase in pH (*p* > 0.05). Significant increase in salivary flow (*p* < 0.01) - No adverse effects
Nascimiento, et al., 2013 ** USA [66]	38 participants (mean age: 26.2 ± 7.8 years old) with or without caries-active	Tooth brushing twice a day for 1 min for 4 weeks: - G1: a fluoride-free toothpaste containing 1.5% arginine (Arg), and (F) Crest Cavity Protection^®^ (1100 ppm F as NaF)	Placebo: - G2: Fluor (F) Crest Cavity Protection ^®^ (1100 ppm F como NaF)	- Arginolytic capacity of biofilm and unstimulated saliva (arginine deiminase system activity -ADS- by quantification of the ammonia) - Quantification of the cariogenic species *Streptococcus mutans* (gtfB gene) and two arginolytic species, *Streptococcus sanguinis* (sagP gene) and *Streptococcus gordonii* (arcA gene) - The Human Oral Microbe Identification Microarray (HOMIM)	- G1: No significant increase in ADC activity in biofilm of the caries-free participants (*p* > 0.05). Significant increase in ADS activity in biofilm of the caries-active participants (*p* < 0.05). Shift in bacterial composition of biofilm microbial profile of caries-active subjects similar to caries-free subjects - G2: Significant increase in ADS activity in biofilm of the caries-free participants (*p* < 0.05)
Li et al., 2015 ** China [67]	5669 children aged 7–12 years old	Tooth brushing twice a day, for two years: - G1: 1.5% Arginine, 1450 ppm F, Dicalcium Phosphate G2: 1.5% Arginine, 1450 ppm F, Calcium Carbonate	Positive control: - G3: 1450 ppm F, Silica	- DMFT scores - Permanent teeth (decayed, missing, filled surfaces -DMFS- scores	- G1: reduction in DMFT, and DMFS. Best reduction compared with G3 - G2: reduction in DMFT, and DMFS. Best reduction compared to G3 - No adverse effects
Xue et al., 2017 ** China [68]	12 participants (mean age: 22.5 ± 2.6 years old) with or without clinical evidence of caries (DMFT ≥ 6)	Tooth brushing twice a day for 3 min, for 2 weeks (crossover study) - G1: arginine-toothpaste (containing 8% arginine and 1450 ppm F−as sodium fluoride)	Placebo: - G2: arginine-free toothpaste (containing 1450 ppm F−as sodium fluoride)	Biofilm: - Lactic acid production - 3-(4,5-dimethylthiazol-2-yl)-2,5-diphenyltetrazolium bromide) metabolic assay - Imaging (scanning electron microscopy (SEM)]	- G1: significant decrease in lactic acid production (*p* < 0.01). No significant changes in metabolic activity. No significant decrease in biofilm biomass or the live/dead bacteria ratio
Razeghian-Jahromi et al., 2022 ** Iran [69]	54 participants aged 15–30 years old	Tooth brushing twice a day for 30 days: - G1: arginine group (8% arginine and 1450 ppm F^−^)	Placebo: - G2: fluoride group (1450 ppm F^−^)	- Relative quantification of Sm (qPCR) (Biofilm)	- G1: Significant decrease of Sm (*p* < 0.05) - G2: No significant decrease of Sm (*p* > 0.05) - No adverse effects
Intervention: Propolis					
Tulsani et al., 2014 India [70]	30 children aged 8–11 years old (mean age 9.13 ± 1.04), with dmft/DMFT ≥ 3	For 15 min: - G1: Propolis chewing gum (6.4% of propolis) - G2: Xylitol chewing gum (15% of Xylitol)	No control/placebo group	- Quantification of Sm (MSA + bacitracin) (saliva)	- The total number of Sm was significantly reduced when compared to baseline in both the groups (*p* < 0.001) - G1: significant reduction in Sm as compared to G2 (*p* < 0.001). - G2: preferred taste compared to group G2
Rodrigues et al., 2020 ** Brazil [71]	24 oral healthy children aged 3–5 years old, with high risk of caries.	Application of varnish to second deciduous molars: - G1: extract of Brazilian red propolis -BRP-(1%) - G2: extract of BRP (2.5%) - G3: extract of BRP (5%) - G4: extract of BRP (10%)	No control/placebo group	- Quantification of Sm (MSA + bacitracin) (stimulated saliva)	- G1 and G2: Significant decrease in Sm (*p* < 0.05) - G2: presented the best results. - No adverse effects
El-Allaky et al., 2020 ** Egypt [72]	60 children aged 6–8 years old, with high caries risk	Twice a day, for 2 weeks: - G1: Propolis chewing gum (2%), for 20 min - G2: 10-mL propolis mouthwash (2%), for 1 min	No control/placebo group	- Biofilm index (O’Leary, Drake & Naylor Plaque Control Record) - Total microorganisms (Blood agar) - Quantification of Sm (MSA) - Children’s preference	- G1 and G2: significant decrease in the biofilm index (*p* < 0.01), with no difference between the two groups (p>0.05) - G1 and G2: significant decrease in Sm (*p* < 0.001), with no difference between the two groups (*p* > 0.05) - Children preferred chewing gum
Rodrigues et al., 2021 ** Brazil [73]	75 children aged 36–71 months with high caries risk	Varnish was applied to the second deciduous molars at three time points: baseline (day 1), at 90 days, and 180 days after treatment initiation: - G1: extract of BRP (2.5%)	Controls: - G2: -CHX- varnish (1%) - G3: fluoride varnish (5%)	- Dental examination (presence or absence of caries lesions ICDAS II) - Quantification of Sm (MSA + bacitracin) (stimulated saliva)	- G1: significant decrease in Sm (*p* < 0.05). At the end: one child with caries - G2: significant decrease in Sm (*p* < 0.05). At the end: 5 children with caries - G3: significant decrease in Sm (*p* < 0.05). No caries lesion developed
Bapat et al., 2021 ** India [74]	120 participants aged 18–20 years old	Rinse twice a day/daily for 1 min with 10-mL mouthwash ( for 3 months: - G1: hot ethanolic propolis extract - G2: cold ethanolic extract	Positive control: - G3: CHX (0.2%) Negative control: - G4: distilled water	- Biofilm index and gingival index/score (Silness y Löe) - Quantification of Sm (MSA + bacitracin) (stimulated saliva)	- G1, G2, G3: significant decrease in biofilm and gingival score (*p* < 0.05). - G4: No effect on biofilm and gingival index
Intervention: Apple					
Rubido et al., 2018 Spain [75]	20 oral healthy participants aged 20–25 years old	Crossover study: - G1: Chewing an apple (Golden Delicious variety)	Control: - G2: Manual tooth brushing without toothpaste.	- Biofilm index (Turesky modification of the Quigley-Hein plaque index) - Analysis of bacterial vitality in unstimulated saliva samples (epifluorescence microscopy technique)	- Tooth brushing produced a significantly lower biofilm index value after cleaning than chewing an apple (*p* < 0.001) - Significant reduction in bacterial vitality immediately afterward in apple and toothbrushing interventions (*p* < 0.001) - Significant increase in bacterial vitality 24 h after chewing an apple compared with that after brushing your teeth (*p* < 0.01).
Mojarad et al., 2021 Iran [76]	48 oral healthy participants aged 20–25 years old	For 2 weeks: - G1: Chewing a yellow apple (160-g) - G2: Chewing a red apple (160-g)	Control: - G3: Manual tooth brushing without toothpaste.	- Biofilm index (O’Leary index)	- Significant reduction in biofilm formation after apple and toothbrushing interventions (*p* < 0.001) - Similar results for apple chewing interventions compared to brushing interventions (*p* > 0.05)—Slightly lower plaque index after chewing a yellow apple
Intervention: Phosphopeptide-Amorphous Calcium Phosphate					
Padminee et al., 2018 India [77]	20 participants aged 18–25 years old (mean age 21.1), with DMF ≥ 3	Chewing gum for 5 min, three times a day, for 2 weeks: - G1: xylitol gum - G2: casein phosphopeptide amorphous calcium phosphate (CPP-ACP) gum	No control/placebo group	Unstimulated saliva: - pH (pH meter) - Buffer capacity - Quantification of Sm (MSA) (stimulated saliva)	- G1 and G2: Significant decrease in Sm, improvement in salivary pH, and buffer capacity (*p*<0.01) - G2: after 24 h and at the end of 2 weeks, more statistically significant improvement in pH than G1 (*p* < 0.05) - No adverse effects
Philip et al., 2019 ** Australia [78]	72 participants aged 10 years old with fixed orthodontic treatment	Tooth brushing twice a day for 5 or 6 weeks: - G1: CPP-ACP (10%) - G2: CPP-ACP with cranberry extract (0.25%)	Positive control: G3: Standard fluoride dentifrice	- Biofilm bacteria identification: 8 caries-associated bacterial species and 6 health associated commensal bacterial species (qPCR)	- G1: significant decrease in Sm and increase in Corynebacterium durum and S. sanguinis compared to G3 (*p* < 0.05) - G2: significant decrease in Sm and *Veillonella parvula* and increase in *Neisseria flavescens, S. sanguinis* compared to G3 (*p* < 0.05) - No difference between G2 and G3
Comparison of Different Extracts					
Mishra et al., 2019 ** India [79]	80 children aged 8–15 years old an increased risk of dental caries with DMFT/dmft > 4	Rinse with 10-mL once a day for 15 days: - G1: Punica granatum seed extracts - G2: Terminalia chebula seed extract - G3: Vitis vinifera seed Extracts	- G4: CHX (0.2%)	- Biofilm index (Turesky–Gilmore–Glickman modification of the Quigley-Hein Plaque Index”) Unstimulated saliva: - Quantification Sm (MSA + bacitracin) - pH value (GC Salivary Kit) - Buffering capacity (titration method)	- G1: maximum Sm count reduction but without significant difference between the other seed extracts groups. Reduction in biofilm. Increase buffer capacity - G2: reduction in Sm count. Reduction in biofilm. Increase in buffer capacity - G3: reduction in biofilm. Reduction in Sm. Reduction in biofilm. Increased buffer capacity - G4: significant decrease of pH. Reduction in biofilm. Increase in buffer capacity
Intervention: Dairy Products					
Somaraj et al., 2018 India [80]	60 oral healthy participants aged 18–24 years old (20.97 ± 1.84 years)	Single portion consumption: - G1: 10-g cube of paneer	Control: - G2: 10-g cube of regular cheese	Unstimulated Saliva: - pH (pH electrode, pH strip and pen pH meter) - Calcium Concentration (colorimetric analysis)	- Significant increase in pH at 15 and 30 min in G1 and G2 (*p* < 0.05) - Significant increase in calcium concentration in both cheeses (*p* < 0.001), higher salivary calcium concentration in G1 (*p* < 0.001)
Intervention: Vitamin D					
Gyll et al., 2018 ** Sweden [81]	85 children aged 8 years old (from basic intervention study DViSUM)	When the children were 6 years old, vitamin D3 was administered daily in a milk-based supplement for 3 months: - G1: 25-μg per day group - G2: 10-μg per day group	Placebo: - G3: 2-μg per day	- Caries status (dmf) - Enamel defects scores (criteria defined by the Commission on Oral Health, Research & Epidemiology) - Serum 25(OH)D and vitamin D-related components, i.e., calcium, phosphate, magnesium, parathyroid hormone (PTH), alkaline phosphatase (ALP), and osteocalcin in Plasma - LL37 levels were analyzed using the (ELISA kit)	- dfs/DFS scores did not differ significantly between the three vitamin D intervention groups (*p* > 0.05) - Higher baseline vitamin D levels were significantly associated with less caries [OR (95% CI) 0.961 (0.929, 0.995; *p* = 0.024)] - Mean LL37 levels were lower in children with insufficient serum vitamin D status than in children with serum 25(OH)D levels ≥ 50 nmol/L after the 3-month intervention [1.09 (0.87, 1.30)] and [2.38 (1.77, 2.99), respectively; *p* < 0.001]
Arponen et al., 2022 Finland [82]	123 healthy infants aged 6–7 years old (mean age: 7.4 years old)	Daily for 2 years: - G1: 30 µg/day vitamin D supplementation - G2: 10 µg/day vitamin D supplementation	No placebo/control group	Oral examination: - Tooth enamel defect (DDE) - Caries finding: dmft/DMFT score - Visible biofilm	- No associations were found between vitamin D intervention group in infancy and oral health or the presence of DDE - The dmft/DMFT score was not different between the intervention groups - 94% of children were vitamin D sufficient (25(OH)D ≥ 50 nmol/l), and 88% had caries-free teeth. 20% had visible biofilm and debris accumulation
Comparison of Different Components Probiotic vs. Green Tea					
Kamalaksharappa et al., 2018 India [83]	40 oral healthy children aged 6–8 years old	Rinse for 1 min once a day for 1 month: - G1: 1-g probiotic in 10-mL distilled water (1.25 billions of *L. acidophilus, L. rhamnosus, Bifidobacterium longum, Saccharomyces boulardii*). -G2: green tea (2-g green tea dip bag dipped in 100-mL warm water for 5 min).	No placebo/control group	- Salivary pH (GC pH strips) (unstimulated whole saliva)	- G2: Significant increase in pH (*p* < 0.001) - G1: Non-significant increase in pH (*p* > 0.05)
Manikandan et al., 2020 India [84]	60 oral healthy children aged 6–8 years old	Rinse for 1 month: - G1: 1-g probiotic in 10 mL of distilled water (1.25 billions of *L. acidophilus, L. rhamnosus, B. longum, Saccharomyces boulardii*) - G2: green tea (2-g green tea dip bag dipped in 100-mL warm water for 5 min)	No placebo/control group	- Salivary pH (GC pH strips) (unstimulated whole saliva)	- G1: significant increase in pH (*p* < 0.001) - G2: significant increase in pH (*p* < 0.001). pH was higher compared to G1
Shetty et al., 2021 India [85]	45 healthy children aged 4–12 years old	Rinse for 1 month: - G1: 1-g probiotic in 10-mL distilled water (1.25 billions of *L. acidophilus, L. rhamnosus, B. longum, S. boulardii)* - G2: green tea (2-g green tea dip bag dipped in 100-mL warm water for 5 min)	- G3: distilled water	- Unstimulated salivary pH after chocolate bar (pH strips)	- G1: Significant increase in pH (*p* < 0.001) - G2: Significant increase in pH (*p* < 0.001) - G3: Decrease in pH
Milk vs. Green Tea					
Talreja et al., 2019 India [86]	30 children aged 8–12 years old	Rinse for 2 min: - G1: Milk with sugar (5%) - G2: Green tea (2%) with sugar (5%)	- G3: Sucrose solution (10%)	- pH value (biofilm)	- G1: Significant increase in pH at 30 min (*p* < 0.05) - G2: Significant increase in pH at different time intervals (*p* < 0.05). Best pH result achieved - G3: Decrease in pH at different intervals. The worst pH result was achieved
Cuasi-Experimental Studies					
Intervention: Probiotic					
Natassa et al., 2019 Indonesia [87]	60 participants aged 12–15 years old, with a minimum of 2–5 surface cavities	Once daily for 7 days: - G1: 180-mL probiotic milk (PM)	- G2: 180-mL non-probiotic milk (NPM)	Unstimulated saliva: - Salivary pH (digital pH meter) - Quantification of Sm (Tryptone Yeast Cystine + sucrose + bacitracin TYCSB media)	- G1: significant increase in pH and significant decrease in Sm (*p* < 0.05). More effective in decreasing Sm (p<0.05) - G2: significant increase in pH and significant decrease in Sm (*p* < 0.05)
Patil et al., 2021 India [88]	10 participants aged 7–12 years old, with dmft/DMFT score of 2–5 (medium risk)	- G1: Topical application of a probiotic solution (*L. acidophilus, L. rhamnosus, L. casei, L. bulgaricus, L. plantarum, B. longum, Streptococcus thermophiles*) to all surfaces of the teeth for a period of 6 days	No placebo/control group	- Quantification of Sm (MSA + bacitracin) (biofilm)	Significant decrease of Sm (*p* < 0.05)
Intervention: Quercetin (Plant Flavonol)					
Ferrazzano et al., 2016 Italy [89]	32 participants with good general health (mean age: 16.3 years old)	First trial: For 30 min: - G1: Quercetin gum (5 mg) - G2: Quercetin gum (10 mg) Second trial: Three times a day for 14 days: - G1: Quercetin gum (10 mg)	First trial: No placebo/control group Second trial: - G2: Placebo: non-quercetin gum	First trial: -Saliva flow rate and salivary pH (pH meters) (unstimulated saliva) - Quercetin content saliva (colorimetric method) Second trial: - Quantification of Sm (MSA + bacitracin)	Fist trial: - G1: increase in saliva flow and pH - G2: increase in saliva flow and pH. Higher quercetin release compared to G1 Second trial: - G1: significant decrease in Sm (*p* < 0.01). - G2: no significant decrease in Sm.
Intervention: Fennel Seeds					
Manohar et al., 2020 India [90]	30 participants over aged over 18 years old with full dentition	Chewing for 5 min: - G1: 1.3 g of fennel seed	No placebo/control group	- Salivary pH (pH strips)	- G1: significant increase in pH (*p* < 0.001)
Intervention: Dairy Product					
Lorenzini, et al., 2022 Italy [91]	9 oral-healthy adults (mean age: 36 ± 4 years old)	For 5 days: - G1: single portion (25 gr) of Italian hard cheese Grana Padano	No placebo/control group	- Bacterial identification (16S rRNA-gene) - Salivary pH (pH strips) (unstimulated saliva)	- G1: significant increase in pH. Reduction in the overall amount of acidophilic bacteria. The Sm/*S. sanguinis* ratio lowers after 5 days.
Intervention: Bee Honey					
Silva, et al., 2025 Brazil [92]	85 adolescents (13 years old)	Chewing one candy for 1 min G1: candy formulated with organic honey. G2: candy formulated with honey rich in antibacterial agents. G3: candy formulated with honey produced by the stingless bee	No control/placebo group	- Quantification of total bacteria (BHI agar) (unstimulated saliva)	- G1, G2 and G3: significant decrease in total bacteria (*p* < 0.05). G2: showed the greatest reduction.
Plant Extracts					
Intervention: Cherry					
Homoki et al., 2018 ** Hungary [93]	70 participants (mean age: 31.2 ± 22.6–58.5 ± 49.4 years old)	Chewing gum for 1 min. - G1: sour cherry extract (Geminis T BHA gum base (CAFOSA), xylitol, citric acid, glycerol, saccharine (Sigma), peppermint volatile oil, and Kirsch Aroma (Akras) (0.1-g anthocyanin rich sour cherry extract)	- G2: control gum products (the sour cherry extract was not added)	Unstimulated saliva: - Measurement of the α-amylase activity (sAA) (Kinetic experiments) - Quantification Sm (trypticase soya base agar enriched with 5% defibrinated sheep blood)	- G1: sAA and Sm levels decreased earlier. Inhibited the human salivary α-amylase enzyme, delaying the starch degradation in the oral cavity
Intervention: Licorice					
Chen et al., 2019 ** China [94]	37 high-risk children (mean age: 4 years old and 10 months) with > 5 × 10^5^ Sm cells per mL saliva	Twice a day for 3 weeks: - G1: sugar-free herbal lollipop containing licorice extracts (10 mg/10 g) + oral health care counseling	- G2: oral healthcare counseling	Unstimulated saliva: - Quantification Sm (specie-specific monoclonal antibody) - Sequencing and analysis (16S rRNA gene)	- G1: Significant reduction in Sm (>80% reduction in bacterial count). May preserve the diversity of the oral microbiome. Had little effect on overall community composition
Intervention: Beetroot					
Sterzenbach et al., 2023 ** Germany [95]	8 healthy participants aged 23–46 years old	Once a day for 15 days (crossover study) - G1: 50-mL commercially beetroot juice (nitrate content of 16 mM or 10 g/l NO3-)	Control: - G2: CHX (0.2%)	Unstimulated saliva and biofilm: - pH value - Determination of Lactate after rising rinsing with apple juice (kit) - Nitrate and Nitrite Measurements (nitrate/nitrite assay kit) - Glucose Assay (D-glucose/D-fructose UV test)	- G1: significant increase in salivary nitrate and nitrite (*p* < 0.05). No significant increase in biofilm nitrate and nitrite. No effect on bacterial salivary lactate production or salivary and biofilm pH
Intervention: Lingonberry					
Pärnänen et al., 2023 ** Finland [96]	21 participants aged 28–91 years old (mean age: 64 years)	Rinse for 30 s, for 6 months: -G1: 10-mL commercial fermented lingonberry juice (0.212% *p*/*v* of polyphenol, 3% *p*/*v* of natural sugars)	No control/placebo group	- DMFT, DMFS, and decayed surfaces (DS) indexes - Probing pocket depths (PPDs), bleeding on probing (BOP%), and visible plaque index (VPI) scale (0–3) Saline rinse samples: - Quantification Sm (MSA + bacitracin), Candida (CHROMagar), and Lb (MRS)	- G1: significant decrease in Sm and *Candida* counts (*p* < 0.05). Significant decrease in DS, BOP, and VPI (*p* < 0.05). Significant increase in Lb (*p* < 0.05). PPDs were not affected
Comparison of Different Components					
Gul et al., 2018 Turkey [97]	10 oral healthy participants aged 20–25 years old, with salivary Sm and Lb levels below 10^5^ CFU/mL and normal buffer capacity	Performed for 1 min: - G1: White cheese - G2: Xylitol chewing gum (content: 15.36%) (chewed for 1 min) - G3: Black Tea solution (10 g/1 L) - G4: Sucrose + White cheese - G5: Sucrose + Xylitol chewing gum - G6: Sucrose + Black Tea solution	- G7: 10% sucrose solution	- Biofilm pH	- G1 and G2: significant increase in pH at different time intervals (*p* < 0.01) - G3: non-significant increase in pH (*p* > 0.05) - G4, G5, G6: pH values decreased following sucrose uptake, they generally increased above the baseline pH within 20 min after the test groups were taken - G7: significant decrease (*p* < 0.001)
A Cross-Sectional Analytical Study					
Author/Country	Population	Group Name		Outcome Measure Techniques	Results
Wang et al., 2022 China [98]	150 children aged 2–5 years old	- G1: early childhood caries (ECC) - G2: severe early childhood caries (S-ECC) - G3: caries-free group		- Dental examination (ICDAS-II, dmfs) - Twenty-four hour dietary intake	- The risk of S-ECC was significantly decreased by vegetables score (OR = 0.137)
Cohort studies					
Author/Country	Population	Exposed Group	Unexposed Group	Outcome Measure techniques	Results
Suárez-Calleja et al., 2021 Spain [99]	188 children aged 6–10 years old (from the INMA-Asturias birth cohort with a dental examination performed)	Vitamin D sufficient level: - G1: above 30 ng/mL (75 nmol/L)	Vitamin D deficient: - G2: below 20 ng/mL (50 nmol/L) Vitamin D Insufficient: - G3: between 20 and 30 ng/mL (50–74 nmol/L)	- Concentration 25(OH) D (blood sample from the mother at 12 weeks of gestation, and from the children at 4 and 8 years of age) - Dental examination (DMFT) - Questionnaires (Diet, nutritional and oro-dental hygiene habits)	- The risk of caries practically tripled when 25(OH) D values were < 20 ng/mL (in both mother and child [OR_gest_ = 2.51(1.01–6.36) and OR_8years_ = 3.45 (1.14–11. 01)]
Chankanka et al., 2015 USA [100]	377 children aged 36–60 months old (from the longitudinal Iowa Fluoride Study)	- G1: high milk consumption at mealtimes	- G2: Less milk consumption at mealtimes	- Three-day diet diaries - Dental examination (Criteria of Pitts): (a) non-cavitated caries, (b) cavitated and/or filled caries; (c) both cavitated and non-cavitated caries 4) the caries-free group	-G1: significantly more likely to be in the caries-free group than in the non-cavitated caries group
Lempert et al., 2015 Denmark [101]	749 children (9 years old) and 340 adolescents (15 years old) (from the Danish EYHS)	- G1: high intake of dairy products, milk, dairy calcium whey, or casein	- G2: Low intake of dairy products, milk, dairy calcium whey or casein.	- Dietary intake: 24-h dietary recall method - Dental examination (sum of DMFT or DMFS in the permanent dentition)	- The risk of caries: a statistically significant inverse association was found about milk intake, as well as between calcium, whey and casein intake.

** Supplementary manual search. G: Group; CFU: Colony Forming Unit; Sm: Streptococcus mutans; ICDAS: International Caries Detection and Assessment System; MSA: Mitis salivarius Agar; Lb: Lactobacillus; MRS: De Man Rogosa Sharpe Agar; SIgA: Secretory IgA; PI: Biofilm index; GI: Gingival index; qPCR: quantitative real-time PCR; DMFT: decayed, missing, filled teeth; deft: decayed, extracted, filled teeth; CHX: Chlorhexidine.

## Data Availability

All data generated or analyzed during this study are included in this published article and its Supplementary Information files.

## Supplementary Materials

The following supporting information can be downloaded at: https://www.mdpi.com/article/10.3390/dj13110518/s1, Table S1: PRISMA 2020 Checklist.

## Appendix A

**Table A1:** Search strategy in various scientific databases.

Scientific Databases	Search Strategy
Pubmed	((“Anticariogenic” [Title/Abstract] OR “Caries-free” [Title/Abstract] OR “Dental Caries Susceptibility” [MeSH Terms] OR “Cariogenic Agents” [MeSH Terms]) AND (“Food” [MeSH Terms] OR “Foods” [Title/Abstract] OR (“Beverages” [MeSH Terms] OR “Beverage” [Title/Abstract]) OR (“Meals” [MeSH Terms] OR “meal *” [Title/Abstract] OR “dinner *” [Title/Abstract] OR “supper *” [Title/Abstract]) OR “Lunch”[MeSH Terms] OR (“Snacks”[MeSH Terms] OR “snack *” [Title/Abstract]))) AND (y_10 [Filter])
Scopus	(TITLE-ABS-KEY (food OR foods OR beverages OR beverage OR lunch OR dinner OR extract) AND TITLE-ABS-KEY (anticariogenic OR “cariostatic agent” OR “Cariogenic Agents”) AND NOT TITLE-ABS-KEY (animals OR cancer OR vitro)) AND PUBYEAR > 2012 AND PUBYEAR < 2024
Ovid	(Food.sh. or Foods.ab,kf,ti. or (Beverages.sh. or Beverage.ab,kf,ti.) or (Meals.sh. or Dinner.ab,kf,ti.) or (Snacks.sh. or Snack.ab,kf,ti.)) and (Anticariogenic.ab,kf,ti. or Cariostatic Agent.sh,ab,kf,ti. or Cariogenic Agent.sh,ab,kf,ti.)
J-Stage	(title:(Food * OR Beverage * OR Dinner * OR Lunch * OR Snack *) OR abstracttext:(Food * OR Beverage * OR Dinner * OR Lunch * OR Snack * OR Extract *) OR keyword:(Food * OR Beverage * OR Dinner * OR Lunch * OR Snack *)) AND (title:(Anticariogenic OR Cariostatic OR Cariogenic OR Antimicrobial OR Bactericide OR Bacteriostatic) OR abstracttext:(Anticariogenic OR Cariostatic OR Cariogenic OR Antimicrobial OR Bactericide OR Bacteriostatic) OR keyword:(Anticariogenic OR Cariostatic OR Cariogenic OR Antimicrobial OR Bactericide OR Bacteriostatic)) NOT (title:(vitro OR Animals OR Situ OR Fertilizer OR Cancer OR Pesticide OR Electric OR Foodborne) OR abstracttext:(Animals OR Situ OR Fertilizer OR Cancer OR Pesticide OR Electric OR Foodborne))
BVS	(mh:(food)) OR (foods) OR (mh:(beverages)) OR (lunch) OR (dinner) OR (mh:(snacks)) OR (snack) AND (anticariogenic) OR (mh:(“Cariostatic Agent”)) OR (mh:(“Cariogenic Agent”)) AND NOT (mh:(animals)) AND NOT (ti:(vitro)) AND NOT (situ) AND NOT (rats) AND (fulltext:(“1” OR “1”) AND type_of_study:(“observational_studies” OR “clinical_trials” OR “incidence_studies” OR “evaluation_studies” OR “systematic_reviews” OR “screening_studies” OR “sysrev_observational_studies”) AND la:(“en” OR “es” OR “pt” OR “fr”)) AND (year_cluster: [2013 TO 2023])
Google Scholar	Anticariogenic OR Cariostatic Food OR Foods OR Meals OR Beverages OR Beverage OR Lunch OR Dinner OR Snack-animals-“in vitro”-“vitro”-“in situ”-electromagnetic-ultrasound-nano-preservative-electric-peroxide-pesticide-fertilizer -foodborne–irradiation

* In some keywords signals truncation to capture both singular and plural (and other variant) forms.

## Appendix B. Quality of Included Studies

**Table A2:** Randomized controlled trials.

Authors	Q1	Q2	Q3	Q4	Q5	Q6	Q7	Q8	Q9	Q10	Q11	Q12	Q13	JBI	GRADE
Poureslami et al., 2013 [25]														12/13	MODERATE
Taipale et al., 2013 [26]														13/13	HIGH
Chinnappa et al., 2013 [27]														4/13	VERY LOW
Pinto et al., 2014 [28]														13/13	HIGH
Nishihara et al., 2014 [29]														8/13	MODERATE
Stensson et al., 2014 [30]														13/13	HIGH
Bhalla et al., 2015 [31]														9/13	LOW
Cortés et al., 2015 [32]														9/13	VERY LOW
Srivastava et al., 2016 [33]														10/13	MODERATE
Koopaie et al., 2019 [34]														10/13	LOW
Piwat et al., 2020 [35]														13/13	HIGH
Ferrer et al., 2020 [36]														13/13	HIGH
Sakhare et al., 2021 [37]														10/13	LOW
Sandoval et al., 2021 [38]														8/13	MODERATE
Janiani et al., 2022 [39]														10/13	MODERATE
Hasslöf et al., 2022 [40]														13/13	MODERATE
Staszczyk et al., 2022 [41]														10/13	MODERATE
Pørksen et al., 2023 [42]														13/13	HIGH
Pørksen et al., 2023 [43]														13/13	HIGH
Campus et al., 2013 [44]														13/13	HIGH
Runnel et al., 2013 [45]														12/13	HIGH
Chi et al., 2017 [46]														13/13	HIGH
Aluckal et al., 2018 [47]														13/13	MODERATE
Abdelwahab et al., 2018 [48]														11/13	MODERATE
Cocco et al., 2019 [49]														13/13	MODERATE
Akgül et al., 2020 [50]														10/13	MODERATE
Neturi et al., 2014 [51]														10/13	MODERATE
Thomas et al., 2016 [52]														11/13	MODERATE
Prihastari and Putri, 2022 [53]														9/13	LOW
Tao et al., 2013 [54]														12/13	HIGH
Hedge and Kamath, 2017 [55]														10/13	LOW
Kamath et al., 2021 [56]														10/13	LOW
Jain et al., 2013 [57]														10/13	LOW
Almaz et al., 2017 [58]														12/13	MODERATE
Kim and Nam, 2021 [59]														13/13	HIGH
Helmy et al., 2021 [60]														13/13	MODERATE
Kamal et al., 2021 [61]														13/13	MODERATE
Kibriya et al., 2023 [62]														7/13	LOW
Bansal et al., 2024 [63]														13/13	HIGH
Salem et al., 2025 [64]														9/13	MODERATE
Vuletic et al., 2013 [65]														12/13	MODERATE
Nascimiento, et al., 2013 [66]														10/13	HIGH
Li et al., 2015 [67]														10/13	HIGH
Xue et al., 2017 [68]														13/13	HIGH
Razeghian-Jahromi et al., 2022 [69]														13/13	HIGH
Tulsani et al., 2014 [70]														11/13	MODERATE
Rodrigues et al., 2020 [71]														8/13	VERY LOW
El-Allaky et al., 2020 [72]														10/13	MODERATE
Rodrigues et al., 2021 [73]														10/13	MODERATE
Bapat et al., 2021 [74]														10/13	LOW
Rubido et al., 2018 [75]														8/13	MODERATE
Mojarad et al., 2021 [76]														7/13	LOW
Padminee et al., 2018 [77]														13/13	MODERATE
Philip et al., 2019 [78]														13/13	HIGH
Mishra et al., 2019 [79]														12/13	MODERATE
Somaraj et al., 2018 [80]														9/13	MODERATE
Gyll et al., 2018 [81]														13/13	MODERATE
Arponen et al., 2022 [82]														13/13	HIGH
Kamalaksharappa et al., 2018 [83]														6/13	LOW
Manikandan et al., 2020 [84]														7/13	LOW
Shetty et al., 2021 [85]														6/13	LOW
Talreja et al., 2018 [86]														6/13	LOW

JBI Critical appraisal checklist for randomized controlled trials. Q1. Was true randomization used for assignment of participants to treatment groups? Q2. Was allocation to treatment groups concealed? Q3. Were treatment groups similar at the baseline? Q4. Were participants blind to treatment assignment? Q5. Were those delivering treatment blind to treatment assignment? Q6. Were outcomes assessors blind to treatment assignment? Q7. Were treatment groups treated identically other than the intervention of interest? Q8. Was follow up complete and if not, were differences between groups in terms of their follow up adequately described and analyzed? Q9. Were participants analyzed in the groups to which they were randomized? Q10. Were outcomes measured in the same way for treatment groups? Q11. Were outcomes measured in a reliable way? Q12. Was appropriate statistical analysis used? Q13. Was the trial design appropriate, and any deviations from the standard RCT design (individual randomization, parallel groups) accounted for in the conduct and analysis of the trial? Green: compliant, Red: non-compliant, Yellow: unclear.

**Table A3:** Quasi-experimental studies.

Authors	Q1	Q2	Q3	Q4	Q5	Q6	Q7	Q8	Q9	JBI	GRADE
Natassa et al., 2019 [87]										7/9	MODERATE
Patil et al., 2021 [88]										2/9	VERY LOW
Ferrazzano et al., 2016 [89]										8/9	MODERATE
Manohar et al., 2020 [90]										4/9	LOW
Lorenzini, et al., 2022 [91]										5/9	MODERATE
Silva et al., 2025 [92]										7/9	LOW
Homoki et al., 2018 [93]										8/9	MODERATE
Chen et al., 2019 [94]										5/9	MODERATE
Sterzenbach et al., 2023[95]										5/9	LOW
Pärnänen et al., 2023 [96]										5/9	LOW
Gul et al., 2018 [97]										6/9	LOW

JBI checklist for quasi-experimental studies. Q1. Is it clear in the study what is the “cause” and what is the “effect” (i.e., there is no confusion about which variable comes first)? Q2. Was there a control group? Q3. Were participants included in any comparisons similar? Q4. Were the participants included in any comparisons receiving similar treatment/care, other than the exposure or intervention of interest? Q5. Were there multiple measurements of the outcome, both pre and post the intervention/exposure? Q6. Were the outcomes of participants included in any comparisons measured in the same way? Q7. Were outcomes measured in a reliable way? Q8. Was follow-up complete and if not, were differences between groups in terms of their follow-up adequately described and analyzed? Q9. Was appropriate statistical analysis used? Green: compliant, Red: non-compliant, Yellow: unclear.

**Table A4:** Analytical cross-sectional studies.

Authors	Q1	Q2	Q3	Q4	Q5	Q6	Q7	Q8	JBI	GRADE
Wang et al., 2022 [98]									7/8	LOW

JBI critical appraisal checklist for analytical cross-sectional studies. Q1. Were the criteria for inclusion in the sample clearly defined? Q2. Were the study subjects and the setting described in detail? Q3. Was the exposure measured in a valid and reliable way? Q4. Were objective, standard criteria used for measurement of the condition? Q5. Were confounding factors identified? Q6. Were strategies to deal with confounding factors stated? Q7. Were the outcomes measured in a valid and reliable way? Q8. Was appropriate statistical analysis used? Green: compliant, Yellow: unclear.

**Table A5:** Cohort studies.

Authors	Q1	Q2	Q3	Q4	Q5	Q6	Q7	Q8	Q9	Q10	Q11	JBI	GRADE
Suárez-Calleja et al., 2021 [99]												11/11	MODERATE
Chankanka et al., 2015 [100]												11/11	MODERATE
Lempert et al., 2015 [101]												11/11	MODERATE

JBI critical appraisal checklist for cohort studies. Q1. Were the two groups similar and recruited from the same population? Q2. Were the exposures measured similarly to assign people to both exposed and unexposed groups? Q3. Was the exposure measured in a valid and reliable way? Q4. We’re confounding factors identified? Q5. Were strategies to deal with confounding factors stated? Q6. Were the groups/participants free of the outcome at the start of the study (or at the moment of exposure)? Q7. Were the outcomes measured in a valid and reliable way? Q8. Was the follow up time reported and sufficient to be long enough for outcomes to occur? Q9. Was follow up complete, and if not, were the reasons to loss to follow up described and explored? Q10. Were strategies to address incomplete follow up utilized? Q11. Was appropriate statistical analysis used? Green: compliant.
