# Supplementary material for: Natural Compounds in Oral Microbiota Modulation and Caries Prevention: A Systematic Review
Source: Dent J (Basel). 2025 Nov 5;13(11):518. doi: 10.3390/dj13110518 (PMC12651059; doi:10.3390/dj13110518)
Supplement: Supplementary file 1 [file dentistry-13-00518-s001.zip › dentistry-3896136-supplementary.pdf]

PRISMA 2020 Checklist

Section and Topic	Item #	Checklist item	Location where item is reported
**TITLE**			
Title	1	Identify the report as a systematic review.	Page 1, line 2
**ABSTRACT**			
Abstract	2	See the PRISMA 2020 for Abstracts checklist.	Page 1, line 16
**INTRODUCTION**			
Rationale	3	Describe the rationale for the review in the context of existing knowledge.	Page 1-2
Objectives	4	Provide an explicit statement of the objective(s) or question(s) the review addresses.	Page 2, line 62-65
**METHODS**			
Eligibility criteria	5	Specify the inclusion and exclusion criteria for the review and how studies were grouped for the syntheses.	Page 3, line 78-84
Information sources	6	Specify all databases, registers, websites, organisations, reference lists and other sources searched or consulted to identify studies. Specify the date when each source was last searched or consulted.	Page 3, line 93-100
Search strategy	7	Present the full search strategies for all databases, registers and websites, including any filters and limits used.	Page 3, line 93-100
Selection process	8	Specify the methods used to decide whether a study met the inclusion criteria of the review, including how many reviewers screened each record and each report retrieved, whether they worked independently, and if applicable, details of automation tools used in the process.	Page 3, line 104-112
Data collection process	9	Specify the methods used to collect data from reports, including how many reviewers collected data from each report, whether they worked independently, any processes for obtaining or confirming data from study investigators, and if applicable, details of automation tools used in the process.	Page 3, line 104-112
Data items	10a	List and define all outcomes for which data were sought. Specify whether all results that were compatible with each outcome domain in each study were sought (e.g. for all measures, time points, analyses), and if not, the methods used to decide which results to collect.	Page 3, line 112-113
	10b	List and define all other variables for which data were sought (e.g. participant and intervention characteristics, funding sources). Describe any assumptions made about any missing or unclear information.	Page 3, line 112-118
Study risk of bias assessment	11	Specify the methods used to assess risk of bias in the included studies, including details of the tool(s) used, how many reviewers assessed each study and whether they worked independently, and if applicable, details of automation tools used in the process.	Page 4, line 121-128
Effect measures	12	Specify for each outcome the effect measure(s) (e.g. risk ratio, mean difference) used in the synthesis or presentation of results.	Page 3, line 110 and 113-114, 116
Synthesis methods	13a	Describe the processes used to decide which studies were eligible for each synthesis (e.g. tabulating the study intervention characteristics and comparing against the planned groups for each synthesis (item #5)).	Page 4, line 114-117
	13b	Describe any methods required to prepare the data for presentation or synthesis, such as handling of missing summary statistics, or data conversions.	Page 3, line 112-113<br>As no meta-analysis was conducted, data preparation focused on extracting and organizing key study characteristics for descriptive

Section and Topic	Item #	Checklist item	Location where item is reported
			synthesis.
	13c	Describe any methods used to tabulate or visually display results of individual studies and syntheses.	Page 3, line 113-116
	13d	Describe any methods used to synthesize results and provide a rationale for the choice(s). If meta-analysis was performed, describe the model(s), method(s) to identify the presence and extent of statistical heterogeneity, and software package(s) used.	Page 3, line 113-116
	13e	Describe any methods used to explore possible causes of heterogeneity among study results (e.g. subgroup analysis, meta-regression).	Page 3, line 111-113
	13f	Describe any sensitivity analyses conducted to assess robustness of the synthesized results.	As no meta-analysis was conducted, formal sensitivity analyses were not performed. However, we assessed the robustness of the narrative findings by comparing results across studies with varying levels of methodological quality. The aim of the review was to identify all components investigated through in vivo studies.
Reporting bias assessment	14	Describe any methods used to assess risk of bias due to missing results in a synthesis (arising from reporting biases).	As no meta-analysis was conducted, formal sensitivity analyses were not performed. However, we assessed the robustness of the narrative findings by comparing results across

PRISMA 2020 Checklist

Section and Topic	Item #	Checklist item	Location where item is reported
			studies with varying levels of methodological quality. The aim of the review was to identify all components investigated through in vivo studies.
Certainty assessment	15	Describe any methods used to assess certainty (or confidence) in the body of evidence for an outcome.	This item could not be fulfilled because most of the studies included in the review did not provide sufficient information to assess the certainty or confidence in the body of evidence for the reported outcomes.
**RESULTS**			
Study selection	16a	Describe the results of the search and selection process, from the number of records identified in the search to the number of studies included in the review, ideally using a flow diagram.	Page 4-5
	16b	Cite studies that might appear to meet the inclusion criteria, but which were excluded, and explain why they were excluded.	Fig 1
Study characteristics	17	Cite each included study and present its characteristics.	Table 2
Risk of bias in studies	18	Present assessments of risk of bias for each included study.	Appendix B.1. Quality of included studies
Results of individual studies	19	For all outcomes, present, for each study: (a) summary statistics for each group (where appropriate) and (b) an effect estimate and its precision (e.g. confidence/credible interval), ideally using structured tables or plots.	Table 2
Results of syntheses	20a	For each synthesis, briefly summarise the characteristics and risk of bias among contributing studies.	Lines 197-201<br>Appendix B.1. Quality of included studies
	20b	Present results of all statistical syntheses conducted. If meta-analysis was done, present for each the summary estimate and its precision (e.g. confidence/credible interval) and measures of statistical heterogeneity. If comparing groups, describe the direction of the effect.	Table 1 and Table 2<br>Although no meta-analysis was

Section and Topic	Item #	Checklist item	Location where item is reported
			conducted, descriptive statistics—including means, medians, standard deviations, and information on statistical significance—were extracted when available
	20c	Present results of all investigations of possible causes of heterogeneity among study results.	Possible causes of heterogeneity were explored by comparing key study characteristics and outcomes across the included studies. Variability was observed in intervention components, dosage and frequency, duration of treatment, population demographics, and types of control groups. These differences may explain the inconsistencies in reported outcomes.
	20d	Present results of all sensitivity analyses conducted to assess the robustness of the synthesized results.	No formal methods were used to assess risk of bias or sensitivity analysis, as all eligible studies identified were

PRISMA 2020 Checklist

Section and Topic	Item #	Checklist item	Location where item is reported
			included in the synthesis. The aim of the review was to identify all components investigated through in vivo studies
Reporting biases	21	Present assessments of risk of bias due to missing results (arising from reporting biases) for each synthesis assessed.	This item was not applicable because no quantitative synthesis or meta-analysis was performed
Certainty of evidence	22	Present assessments of certainty (or confidence) in the body of evidence for each outcome assessed.	This item was not applicable
**DISCUSSION**			
Discussion	23a	Provide a general interpretation of the results in the context of other evidence.	Line 203-297
	23b	Discuss any limitations of the evidence included in the review.	Line 297-306
	23c	Discuss any limitations of the review processes used.	No formal meta-analysis was conducted, which limits the ability to quantify effect sizes or statistically assess heterogeneity”
	23d	Discuss implications of the results for practice, policy, and future research.	Line 307-314
**OTHER INFORMATION**			
Registration and protocol	24a	Provide registration information for the review, including register name and registration number, or state that the review was not registered.	Page 3, line 74-75
	24b	Indicate where the review protocol can be accessed, or state that a protocol was not prepared.	Page 3, line 75
	24c	Describe and explain any amendments to information provided at registration or in the protocol.	Page 3, line 75
Support	25	Describe sources of financial or non-financial support for the review, and the role of the funders or sponsors in the review.	Lines 329-331
Competing interests	26	Declare any competing interests of review authors.	Line 338
Availability of data, code and other materials	27	Report which of the following are publicly available and where they can be found: template data collection forms; data extracted from included studies; data used for all analyses; analytic code; any other materials used in the review.	Lines 334-335

From: Page MJ, McKenzie JE, Bossuyt PM, Boutron I, Hoffmann TC, Mulrow CD, et al. The PRISMA 2020 statement: an updated guideline for reporting systematic reviews. BMJ 2021;372:n71. doi: 10.1136/bmj.n71.
This work is licensed under CC BY 4.0. To view a copy of this license, visit https://creativecommons.org/licenses/by/4.0/
